# Translating short-form Python exercises to other programming languages using diverse prompting strategies

**DOI:** 10.1093/gigascience/giaf149

**Published:** 2025-12-08

**Authors:** Stephen R Piccolo, Harlan P Stevens

**Affiliations:** Department of Biology, Brigham Young University, Provo, UT 84602, USA; Department of Biology, Brigham Young University, Provo, UT 84602, USA; Harvard Medical School, Harvard University, Boston, MA 02115, USA

**Keywords:** bioinformatics education, translating programming languages, large language models, Python programming language, Rust programming language, automated code translation

## Abstract

**Background:**

With the increasing complexity and quantity of experimental and observational data, life scientists rely on programming to automate analyses, enhance reproducibility, and facilitate collaboration. Scripting languages like Python are often favored for their simplicity and flexibility, enabling researchers to focus primarily on high-level tasks. Compiled languages such as C++ and Rust offer greater efficiency, making them preferable for intensive or repeated computations. In educational settings, instructors may wish to teach both types of languages and thus translate content from one programming language to another. In research contexts, researchers may wish to implement their ideas in one language before translating the code to another. However, translating between programming languages requires significant effort, prompting our interest in using large language models (LLMs) for semi-automated code translation.

**Results:**

This study explores the use of an LLM (GPT-4) to translate 559 short-form programming exercises from Python into C++, Rust, Julia, and JavaScript. We used 3 prompting strategies—instructions only, code only, or both combined—and compared the translated code’s output against the Python code’s output. Translation success differed considerably by prompting strategy, and at least one of the strategies tested was effective for nearly every exercise. The highest overall success rate occurred for Rust (99.5%), followed by JavaScript (98.9%), C++ (97.9%), and Julia (95.0%).

**Conclusions:**

Our findings demonstrate that LLMs can effectively translate small-scale programming exercises between languages, reducing the need for manual rewriting. To support education and research, we have manually translated all exercises that were not translated successfully through automation, and we have made our translations freely available.

## Introduction

Due to the growing scale and complexity of biological data, computer-programming skills have become essential for modern life scientists [1]. Researchers use programming to automate tasks such as processing genomic datasets, running simulations, and creating visualizations. Writing code to carry out these tasks promotes reproducibility, enabling other scientists to scrutinize, validate, and extend computational workflows [2]. In turn, reproducibility fosters collaboration and accelerates scientific discovery.

When choosing a programming language, life scientists often consider both their own expertise and the demands of the task [3]. For high-level data analyses, scripting languages like Python and R are widely used [3–5]. These languages make it relatively easy to perform tasks such as importing and exporting data, performing statistical analyses, training machine learning models, and generating graphics [6, 7]. Because such analyses may be specific to a particular study and run only once, speed and resource efficiency are not always priorities. In contrast, tasks that are repeated many times, are computationally intensive, or require fine-grained control over memory often benefit from implementation in compiled languages like C++ or Rust [8]. For example, Burrows-Wheeler Aligner (used for aligning short DNA sequences) [9], Salmon (for transcript quantification) [10], and the Geospatial Data Abstraction Library (for geospatial data processing) [11] are all written primarily in compiled languages to maximize performance.

Scripting and compiled languages differ in their syntax, execution model, and levels of abstraction. In scripting languages, an interpreter executes code line by line. These languages typically feature relatively simple syntax [12] and dynamic typing. They also abstract away complex operations such as memory management, garbage collection, and thread control. As a result, scientists can concentrate on high-level research tasks without needing to manage low-level system details. In contrast, most compiled languages are statically typed and require greater attention to system-level concerns like memory allocation and thread handling. While programming in compiled languages may be more time-consuming and cognitively demanding, the resulting programs generally execute faster and use computing resources more efficiently [12, 13].

In postsecondary educational settings, it is increasingly common for instructors to teach scripting rather than compiled languages in introductory programming courses [14–16]. By learning a scripting language at the outset, students might more easily master programming logic before moving to more advanced topics. However, evidence is mixed regarding whether starting with a scripting or compiled language better prepares students for more advanced programming tasks [17–20]. When students transition from scripting to compiled languages, they often struggle with new syntax, static typing, and manual memory management. To ease this transition, it may be pedagogically useful to have students reimplement familiar exercises—such as those completed previously in a scripting language—as they learn a compiled language. This practice might encourage comparisons between paradigms, reinforce core programming concepts, and reduce the cognitive load associated with learning lower-level programming techniques.

In research settings, scientists may wish to prioritize implementing and testing core logic before focusing on performance, resource usage, or security concerns [21]. This practice—commonly referred to as rapid prototyping—enables researchers to explore ideas and validate functionality quickly. One approach is to write the initial version of a program in a scripting language, where development is faster and more flexible. Once the logic is sound, the code can be translated into a compiled language to improve execution speed or scalability [22]. This workflow allows researchers to balance development efficiency with computational performance.

Human proficiency in multiple programming languages enables researchers to translate code manually from one language to another. However, acquiring and maintaining fluency in multiple languages demands considerable time and energy—resources that could be spent on other research tasks. A promising alternative is to use large language models (LLMs), which can not only generate code from natural language prompts but also translate code between programming languages. Modern LLM chatbots typically support zero-shot prompting, which allows users to obtain meaningful outputs even when the underlying model has not encountered similar examples during training. If these models can perform code translations accurately and with minimal human input, they could accelerate rapid prototyping in research and aid in developing educational materials that help learners transition between programming languages.

To date, most existing research in this area has focused on source-to-source translation [23], translation between compiled languages [24–27], and translation of entire projects [24, 28]. Less attention has been paid to how these models could support transitions between scripting and compiled languages and on comparing prompting strategies.

We evaluated the ability of an LLM (ChatGPT 4) to translate short-form educational programming exercises from Python to 4 target languages: 3 compiled languages (C++, Rust, and Julia) and 1 scripting language (JavaScript). Our dataset consisted of 559 exercises originally written for Python; each includes both English-language instructions and code. Many of the exercises are oriented specifically toward the life sciences. To assess translation accuracy, we used unit-test results as a reference and performed manual reviews when necessary. We compared model performance across different input types and target languages and categorized error types. In addition to summarizing our findings, we introduce a novel educational resource containing validated solutions to all 559 exercises and all 5 programming languages. We anticipate that this resource will be useful for both education and research.

## Materials and Methods

We gathered short-form, Python programming exercises from Austin et al. [29] (*n* = 426) and Piccolo et al. (*n* = 133) [30]. Each exercise came with instructions (directed at students and other learners), an example solution, and Python code to test students’ solutions. We wrote Python code to generate spreadsheets that were structured consistently for the 2 sources. We reviewed each problem and adjusted the wording to improve clarity, make the instructions more consistent across the exercises, and use terminology that was less specific to Python programming. For example, we removed the term “Python” from the instructions and used “vector,” “HashMap,” and “null” instead of “list,” “dictionary,” and “None,” respectively. We removed URLs from the instructions, corrected typographical errors, and altered the example solutions to improve clarity or succintness (in a few cases). We excluded exercises that were incompatible between languages. In particular, some of the Piccolo et al. [30] exercises used the *pandas* and *seaborn* packages for analysis tasks [7, 31], but corresponding packages were unavailable for the programming languages to which the exercises would be translated. In some cases, the example solutions from Piccolo et al. [30] included data files to be used as inputs; we embedded paths to these files within the spreadsheets. When testing the generated code, we copied these files to the current working directory.

For each exercise, we reviewed the Python code and student-directed instructions and assigned a category that reflected the types of programming skills necessary to solve the exercises. These categories and their descriptions are listed in Table 1.

**Table 1:** Exercise categories and descriptions. For each exercise, we reviewed the Python code and student-directed instructions and assigned a category that reflected the types of programming skills necessary to solve the exercises. These are listed in this table.

Term	Description
Basic Data Manipulation [DM]	Tasks involving creating, modifying, filtering, combining, or reorganizing standard data structures such as lists, vectors, tuples, sets, and dictionaries.
String Processing (No Regex) [SP]	Tasks that manipulate plain strings using basic operations such as splitting, joining, trimming, replacing characters, or checking prefixes/suffixes—without using regular expressions.
Regular-Expression String Processing [RX]	Tasks that require pattern matching, extraction, or substitution using explicit regular expressions.
Arithmetic, Numeric Computation, & Number Theory [NC]	Tasks involving numerical calculations or properties of numbers, including sequences, digit manipulations, primes, divisors, summations, and other pure arithmetic logic.
Geometry, Scientific Formulas, & Applied Math [GA]	Tasks using real-world mathematical formulas (e.g., area, volume, physics/biology formulas, unit conversions) where correctness depends on applying the proper equation.
Algorithms & Data Structures [AL]	Tasks requiring explicit algorithmic logic—such as searching, sorting, recursion, dynamic programming, subsequence/subarray algorithms, or other structured computational procedures.
Bitwise Operations, Binary Manipulation, & Low-Level Logic [BW]	Tasks using binary arithmetic or bitwise operators (AND, OR, XOR, shifts) to examine or modify numerical values.
File Processing, Parsing, & Domain-Specific Data Handling [FP]	Tasks that involve reading, writing, or transforming file contents, parsing external data formats, or performing computations based on domain-specific datasets (e.g., biological sequences, tabular data).

Using OpenAI’s Chat Completions API, we evaluated the ability to translate the example solutions and test code for the Python exercises to other programming languages: C++, Rust, Julia, and JavaScript. When invoking the API, we used version “gpt-4-0314” of the model and the default *temperature* setting of 0.7. For each exercise and programming language, we separately evaluated each of 3 input types: (i) the instructions only, (ii) the Python example solution, or (iii) the instructions and the example solution. The API inputs consisted of the programming task instructions and/or Python code (see Data Availability) and expected outputs.

When using only the instructions as the input, we provided the following user and systems messages to the API (with placeholders replaced with actual values for each exercise):

Prompt:

{instructions}

Python testing code (needs to be translated to {otherlanguage}):

{python_test_code}

Expected output (converted to lower case):

{expected_output}

You are a helpful assistant who generates {otherlanguage} code. You are given a prompt and some accompanying Python code for testing. Implement {otherlanguage} code in response to the prompt. Translate the testing code to {otherlanguage} and put it in a main() method. Do not provide any comments on how the code works or *any other* text. Provide *code only*. Surround all code with backticks.

When using only the Python code as the input, we provided the following user and systems messages to the API:

Python code to translate:

{python_code}

Python testing code (needs to be translated to {otherlanguage}):

{python_test_code}

Expected output (converted to lower case):

{expected_output}

You are a helpful assistant who translates Python code to {otherlanguage} code. The second part of the code is testing code. Translate all of the code to {otherlanguage} and invoke it in a main() method. Do not provide any comments on how the code works or *any other* text. Provide *code only*. Surround all code with backticks.

When using the instructions and the Python code in the prompt, we provided the following user and systems messages to the API:

Prompt:

{instructions}

Python testing code (needs to be translated to {otherlanguage}):

{python_test_code}

Expected output (converted to lower case):

{expected_output}

Example implementation of the code in Python:

{python_code}

You are a helpful assistant who generates {otherlanguage} code. You are given a prompt, some accompanying Python code for testing, and an example in Python. Implement {otherlanguage} code in response to this information. Translate the test code to {otherlanguage} and put it in a main() method. Do not provide any comments on how the code works or *any other* text. Provide *code only*. Surround all code with backticks.

In all cases, after receiving translated code from the API, we attempted to compile the code (where applicable) and execute it locally. For C++, we used *Apple clang version 11.0.3 (clang-1103.0.32.59)*. For Rust, we used version 1.67.1 of *cargo*. For Julia, we used version 1.9.2. For JavaScript, we used version 9.8.0 of *npm*. When the generated code required an additional package, we installed it using the relevant package manager.

To facilitate the evaluation process, we generated a spreadsheet for each prompting strategy and programming language. These spreadsheets contain the raw outputs generated by the model, the code parsed from these outputs, the standard output and standard error resulting from code execution, and whether the standard output (converted to lowercase) matched the expected output. In cases where the outputs did *not* match, we reviewed the outputs manually and recorded a high-level reason for the failure to match. In some cases, we deemed that even though the model’s output did not perfectly match the expected output, the outputs were qualitatively identical. Many exercises require objects to be printed to standard output, yet conceptually, identical objects may be represented differently when printed at runtime for different programming languages. For example, the Python interpreter typically displays strings using single quotation marks, whereas the Rust runtime displays strings with double quotation marks. Similarly, Python uses native syntax for floating-point numbers, while Rust prints such numbers with named fields. In our analysis, when a generated solution passed neither automated validation nor manual review, we repeated the process of generating and evaluating code—up to 10 times. The prompts were identical across these iterations, and no new information was provided to the models from previous failed attempts.

In cases where the LLM did not generate a passing solution for a given programming language after 10 attempts, we manually created a solution. After doing so, we executed the code and verified that its output matched the expected output (via automated or manual review).

To perform these analyses, we wrote scripts for Python (version 3.9) and R (version 4.4.1) [32]. Additionally, we used *tidyverse* packages (version 2.0.0) and the *ggupset* package (version 0.4.1) when analyzing the data [6, 33].

## Results

We evaluated an LLM’s ability to translate 559 short-form programming exercises from Python into 4 target languages: C++, Rust, Julia, and JavaScript. Each exercise was translated using 1 of 3 input types: (i) English-language instructions only (modified to avoid Python-specific terminology), (ii) Python code only, or (iii) both instructions and code. We considered a translation to be successful if it produced the expected result within 10 attempts.

Overall, translation was most successful for Rust and JavaScript (Table 2). The highest success rates—96.8% for Rust and 96.2% for JavaScript—were achieved when both instructions and code were provided. However, interestingly, C++ performed best (94.3%) when only the code was given, while Julia achieved its highest success rate (87.8%) with instructions alone.

**Table 2:** Translation success rates after 10 iterations. For each programming language and input type, this table shows the success rate for translating to the target language within 10 iterations.

Target language	Instructions only	Code only	Instructions and code	Any
C++	89.6%	94.3%	92.1%	97.9%
Rust	**92.7%**	95.3%	**96.8%**	**99.5%**
Julia	87.8%	83.0%	85.2%	95.0%
JavaScript	**92.7%**	**95.7%**	96.2%	98.9%

We quantified performance levels when *at least one input type* led to a successful translation per exercise. For example, if the instructions-only prompt led to a successful translation but the code-only prompt did not, we counted this as a success. Using this approach, the model successfully translated 556 of 559 exercises for Rust, yielding an overall success rate of 99.5%. By this measure, success rates reached at least 95.0% for all 4 programming languages (Table 2).

**Figure 1:**
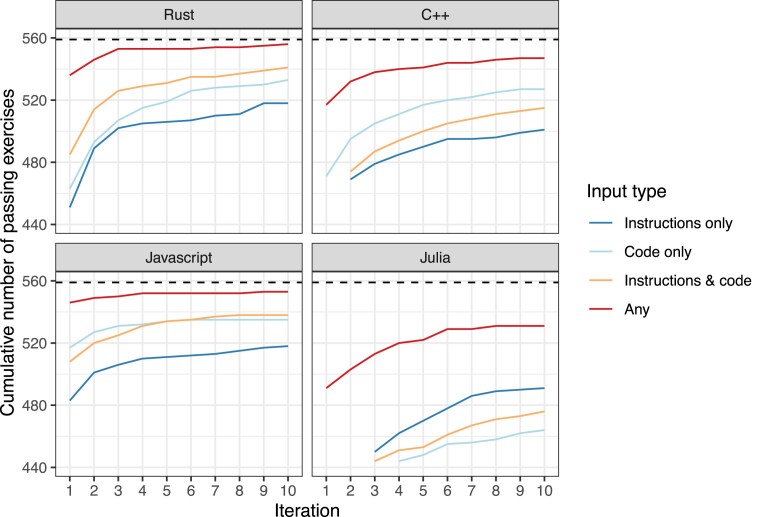
Cumulative number of passing exercises per iteration, input type, and programming language. When an exercise did not pass the tests on the first attempt, we repeated the code generation and validation process for up to 10 attempts. This graph illustrates the cumulative number of exercises that passed the tests as the iterations progressed.

All target languages demonstrated performance improvements over successive iterations, with varying degrees of effectiveness, depending on the prompting strategy employed. The *Any* strategy—indicating at least one successful translation from any of the 3 input types—consistently outperformed individual prompting strategies across all programming languages (Fig. 1). As an example, for Rust translations, the proportion of passing exercises improved rapidly between iterations 1 and 4—increasing by as much as 9.7%—before beginning to plateau. Proportions increased by a maximum of 3.2% between iterations 4 and 10 for Rust translations. Across all languages, there were often diminishing returns after approximately 7 iterations.

**Figure 2:**
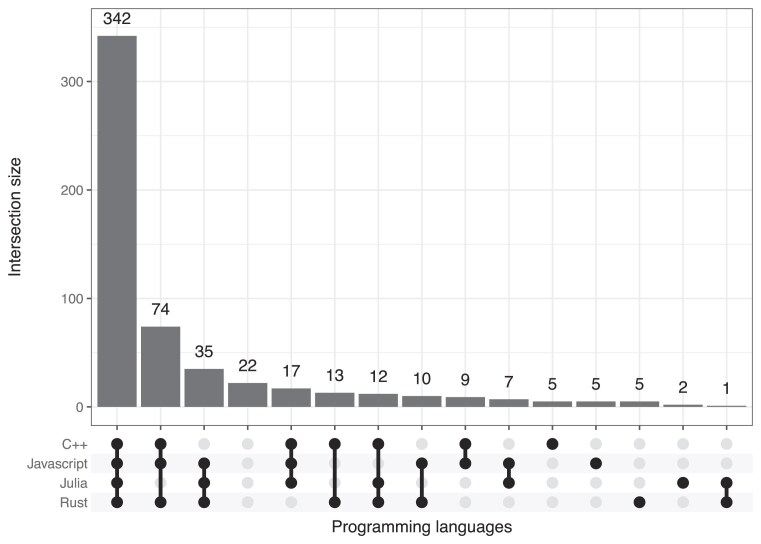
Overlap in translation success among programming languages. For each combination of target programming languages, we counted the number of times that translation was successful across all input types.

**Figure 3:**
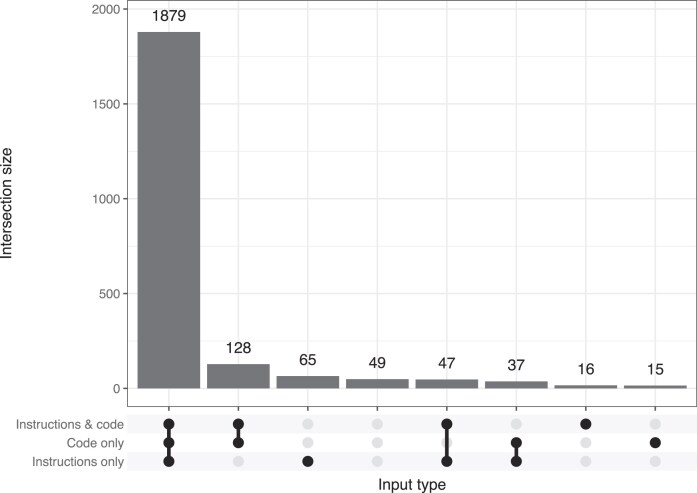
Overlap in translation success among input types. For each exercise and across all 4 target programming languages, we counted the number of times that a translation was successful for each combination of input types.

To provide insight on cross-language similarities and differences, we counted the number of times that translation was successful across *all* input types for each combination of target programming languages (Fig. 2). Most commonly, translation was successful for all 4 languages (61.2% of scenarios). It was also common for 3 of the 4 languages—in different combinations—to be successful (24.7% of scenarios). For 22 (3.9%) of the exercises, translations were successful for none of the programming languages across all input types. Additionally, for each exercise and across all 4 target programming languages, we counted the number of times that a translation was successful for each combination of input types (Fig. 3). Translations were successful for *all* 3 inputs types in 84.0% of scenarios. The most common other scenarios were for translations to be successful either for (i) code only *and* both inputs (5.7%) or (ii) instructions only (2.9%).

**Figure 4:**
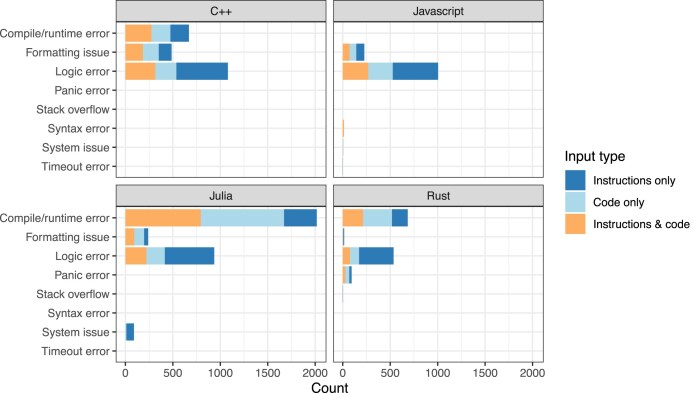
Outcome types for initially nonpassing translated code. Upon compiling and/or executing translated code and finding that the code’s output did not match the expected output for a given exercise, we categorized the reason for this mismatch. This chart summarizes these outcomes across the input types and programming languages.

The sources of errors differed considerably across the programming languages. Most frequently, errors for the generated Rust and Julia code occurred at compile time or runtime (Fig. 4). However, for C++, logic errors were more common than compiling errors. For JavaScript (a scripting language), logic errors were also the most common type. All 4 languages exhibited formatting mismatches—cases where the program output differed from the expected output in minor ways (e.g., spacing or punctuation); formatting errors were most common for the translated C++ code. In many instances, we deemed these differences acceptable upon manual review.

**Figure 5:**
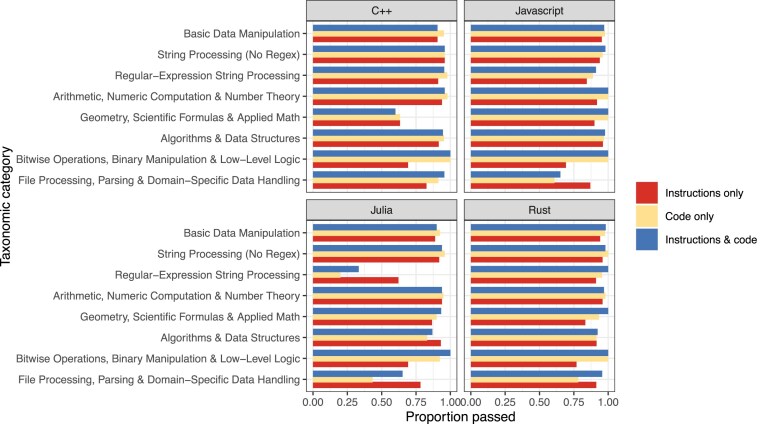
Proportion of exercises passed by taxonomic category. For each target programming language and input type, we calculated the proportion of exercises that were translated successfully per taxonomic category.

**Figure 6:**
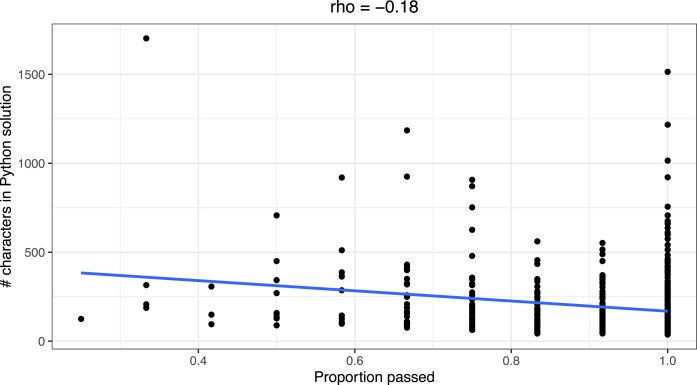
Relationship between code length and translation success. For each exercise, we calculated the number of characters in the Python solution. This plot illustrates the relationship between this measure of code length and the proportion of times that the exercise was translated successfully.

To provide additional insight into factors associated with successful translation, we manually assigned a category to each programming exercise (see Materials and Methods). Across all programming languages and input types, exercises in the “File Processing, Parsing, & Domain-Specific Data Handling” (77.9%) and “Regular-Expression String Processing” (79.3%) categories were least often translated successfully. Exercises in the “String Processing (No Regex)” (95.9%) and “Arithmetic, Numeric Computation, & Number Theory” (96.1%) categories were most often translated successfully. These success rates differed considerably across programming languages and input types (Fig. 5). Using the number of characters in the Python solutions as an indicator of code complexity, we evaluated the relationship between code length and successful code translation. Across all programming languages and input types, there was a statistically significant, negative correlation between code length and translation success (Spearman’s rho = −0.18, *P* = 2.4e-05; Fig. 6). In simpler terms, translations were more successful for relatively short code examples.

**Figure 7:**
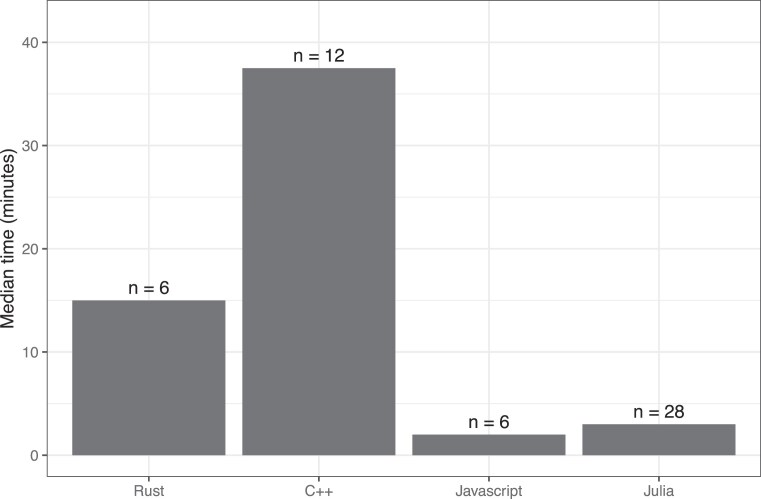
Approximate time to manually write code for exercises that were not successfully translated by the large language model. For exercises that were not successfully translated by the large language model for any of the prompting strategies, we manually created a functional solution. This graph illustrates the median time (in minutes) that we spent on creating solutions for each of the programming languages.

In cases where functional code had *not* been generated for *any* of the input types, we manually wrote functional code, using the generated code as a starting point. During this process, we used OpenAI’s chatbot (ChatGPT 4) as an informal consultant. In each case, we tested the code using the same automated process that we used to validate the generated code. The amount of time it took to write functional code ranged from approximately 1 minute to 3 hours, depending on the exercise and programming language. The median time per exercise was considerably shorter for JavaScript and Julia than for C++ and Rust (Fig. 7).

To support future research and education efforts, we have made all of the source (Python) materials and translated code available for free, both as standalone files (see Materials and Methods) and via our CodeBuddy web application (course name: “Sharpen Your Skills: Exercises in 5 Popular Programming Languages”) [34]. Via CodeBuddy, anyone with an Internet connection can create a free account and attempt to solve the exercises for any of the 5 programming languages.

## Discussion

Researchers have long studied the ability to create statistical models of code structure [35–37] and use machines to translate code from one language to another [36, 38, 39]. In recent years, the field has shifted from rule-based transpilers to mostly machine learning approaches, especially using neural networks [24, 25, 41]. Our work addresses the problem of translating short-form exercises consisting of relatively few (typically under 20) lines of code. Roziere et al. and others have facilitated work in this area by releasing examples of “parallel” solutions in multiple programming languages, making it feasible to train models on aligned examples of functionally equivalent code [23, 40, 41, 42]. Additionally, researchers have emphasized the importance of using unit tests to verify that outputs are equivalent for different implementations of the same logic [42, 43]. LLMs, supervised by humans, may be helpful in creating such tests [25, 44]. Some researchers have sought to translate larger codebases; strategies have included translating one portion at a time independently [26] and using iterative prompting strategies [45]. However, these attempts have been met with varied levels of success [25, 26, 28, 45].

In this study, we have demonstrated that a general-purpose LLM is capable of facilitating semi-automated translation of short-form programming exercises from one programming language to another with relatively little human effort. Our research differs from prior work in multiple ways. Rather than using existing benchmark datasets that may have been used as training inputs for the GPT-4 model, we used exercises that were publicly available in Python. Additionally, we asked the model to translate unit tests from Python to 4 target languages. Prior studies have focused primarily on Python, Java, and C++, although attention has shifted recently to Rust translations due to Rust’s memory safety, particularly in systems programming contexts [24–27]. Life scientists are turning to Rust as a way to improve the speed, safety, and reliability of their computational tools—particularly for tasks that are computationally and data intensive [8]. We included the Julia programming language in our analysis, in part because it combines the expressiveness of high-level languages like Python and R with execution speeds closer to those of C++ and Rust [21]. Of note, Julia is different from C++ and Rust in that it is just-in-time compiled and dynamically typed. Another difference between our work and others’ is that we attempted 3 different prompting strategies and compared the LLM’s ability to translate given these different inputs. Nearly all prior work has either used only instructions as prompts or attempted to translate from code in one programming language to another. Our results demonstrate that translation performance varies by prompting strategy and that these strategies are complementary. Finally, our work differs from prior work in that we (i) manually solved the exercises that were not automatically solved, (ii) made these available for others to solve via a web interface, and (iii) have shared not only the source code and translated code—thus constituting a corpus of parallel examples for 5 languages—but also the full computational workflow we used to perform the analysis.

Whereas Python and JavaScript are interpreted languages, 3 of the target languages (Rust, C++, and Julia) are compiled languages. Differences between programming paradigms provide some insight about whether translation successes and failures stem from language paradigm differences or from other factors in the translation process. Success rates for Rust and JavaScript followed similar patterns to each other, suggesting that language paradigm alone does not account for translation performance. Instead, it points to the possibility that the availability and quality of training data for each language, as well as the model’s exposure to how each language is commonly written and used in practice, play substantial roles.

Although it might be ideal to perform code translations in a fully automated manner, our analyses show that some manual review and translation are necessary for a large corpus of programming exercises. LLMs provide an opportunity to complement human efforts [46] and reduce the overall time and costs involved. However, it is difficult to quantify these savings, which depend on factors like the labor cost of a human translator and the opportunity cost of redirecting humans’ time away from other tasks.

In educational settings, semi-automatic translation may facilitate students’ learning as they progress from one programming language to another. Similarly, it can help instructors adapt to industry trends and other factors that influence the choice of programming language in computational courses. Alternatively, an instructor or learner might wish to translate a compiled-language implementation of a particular algorithm to a scripting language so that its logic is more accessible.

In research settings, semi-automatic translation can enable rapid prototyping, in which logic is first implemented in a scripting language and later optimized for speed and other performance factors. In addition, this capability may take the place of creating interfaces between programming languages [47, 48].

Our study is limited in several ways. The author (H.P.S.) who manually solved the exercises that were not translated automatically was an undergraduate student. He had taken computer science and bioinformatics courses primarily in Python and C++. However, he was new to Rust and Julia and had minimal experience with JavaScript. Accordingly, the time required to manually solve these exercises may not reflect how long it would take a more experienced programmer to complete them—particularly a programmer with deep familiarity across all target languages. Additionally, the LLM we used may perform differently in other contexts. While it showed promise for translating short, self-contained exercises, it may be considerably less effective for more complex tasks, such as translating large projects, interactive notebooks, or domain-specific libraries. While our results broadly demonstrate the promise of LLMs for short-code translation, we only tested translation using GPT-4. While GPT-4 was state-of-the-art at the time of these experiments, newer or more specialized LLMs would likely produce different results. Furthermore, to our knowledge, no translated versions of these datasets were publicly available at the time of our analysis. However, without specific knowledge of the sources used to train this proprietary model, it is impossible to confirm that the model had not previously been exposed to translated versions of the exercises. Another limitation is that we repeated the same prompt in each iteration. While this approach made retries more efficient, a more interactive process incorporating human feedback likely would have reduced the number of retries. One aspect of the process that did involve human interpretation was assessing whether code outputs were qualitatively identical between programming languages; developing automated equivalence checks (perhaps using an LLM) may have reduced the subjectivity of this process.

Important work remains to identify best practices for using LLMs in translation settings. Potential solutions include integrating automated translation directly with human review, developing prompting strategies that improve consistency across files, or training fine-tuned models on curated multilanguage datasets. Additionally, interdisciplinary collaborations between domain experts and language model researchers may be essential to build robust, trustworthy tools for code translation in scientific and educational contexts.

## Supplementary Material

giaf149_Authors_Response_To_Reviewer_Comments_Original_Submission

giaf149_GIGA-D-25-00265_Original_submission

giaf149_GIGA-D-25-00265_Revision_1

giaf149_Reviewer_1_Report_Original_Submission Jacob Durrant -- 8/4/2025

giaf149_Reviewer_2_Report_Original_Submission C. Titus Brown -- 8/10/2025

## Data Availability

An interactive version of the coding exercises can be found on the CodeBuddy website [50]. The scripts, programming task instructions, Python solution code, Python test code, and data files (where relevant) for the exercises, as well as translated solutions for each language, are stored in an open-access repository at Zenodo [51].

## Abbreviations

LLM: large language model.
