# Supplementary material for: Translating short-form Python exercises to other programming languages using diverse prompting strategies
Source: Gigascience. 2025 Dec 8;14:giaf149. doi: 10.1093/gigascience/giaf149 (PMC12758376; doi:10.1093/gigascience/giaf149)
Supplement: giaf149_GIGA-D-25-00265_Original_submission [file giaf149_giga-d-25-00265_original_submission.pdf]

GigaScience

Translating short-form Python exercises to other programming languages using diverse prompting strategies

--Manuscript Draft--

**Manuscript Number:**	GIGA-D-25-00265
**Full Title:**	Translating short-form Python exercises to other programming languages using diverse prompting strategies
**Article Type:**	Research
**Funding Information:**	
**Abstract:**	With the increasing complexity and quantity of data, life scientists rely on programming to automate analyses, enhance reproducibility, and facilitate collaboration. Scripting languages like Python are often favored for their simplicity and flexibility, enabling researchers to focus primarily on high-level tasks. Compiled languages such as C++ and Rust offer greater efficiency, making them preferable for intensive or repeated computations. In educational settings, instructors may wish to teach both types of languages and thus may wish to translate content from one programming language to another. In research contexts, researchers may wish to implement their ideas in one language before translating the code to another. However, translating between programming languages requires significant effort, prompting our interest in using large language models (LLMs) for semi-automated code translation. This study explores the use of an LLM (GPT-4) to translate 559 short-form programming exercises from Python into C++, Rust, Julia, and JavaScript. We used three prompting strategies—instructions only, code only, or both combined—and compared the translated code's output against the Python code's output. Translation success differed considerably by prompting strategy, and at least one strategy was successful for nearly every exercise. The highest overall success rate occurred for Rust (99.5%), followed by JavaScript (98.9%), C++ (97.9%), and Julia (95.0%). Our findings demonstrate that LLMs can effectively translate small-scale programming exercises between languages, reducing the need for manual rewriting. To support education and research, we have manually translated all exercises that were not translated successfully through automation, and we have made our translations freely available.
**Corresponding Author:**	Stephen R Piccolo, Ph.D.<br>Brigham Young University<br>Provo, UT UNITED STATES
**Corresponding Author Secondary Information:**	
**Corresponding Author's Institution:**	Brigham Young University
**Corresponding Author's Secondary Institution:**	
**First Author:**	Stephen R Piccolo, Ph.D.
**First Author Secondary Information:**	
**Order of Authors:**	Stephen R Piccolo, Ph.D.
	Harlan P Stevens
**Order of Authors Secondary Information:**	
**Additional Information:**	
**Question**	**Response**
Are you submitting this manuscript to a special series or article collection?	No
**Experimental design and statistics**	Yes

Powered by Editorial Manager® and ProduXion Manager® from Aries Systems Corporation

Full details of the experimental design and statistical methods used should be given in the Methods section, as detailed in our Minimum Standards Reporting Checklist. Information essential to interpreting the data presented should be made available in the figure legends.<br><br>Have you included all the information requested in your manuscript?	
**Resources**<br><br>A description of all resources used, including antibodies, cell lines, animals and software tools, with enough information to allow them to be uniquely identified, should be included in the Methods section. Authors are strongly encouraged to cite Research Resource Identifiers (RRIDs) for antibodies, model organisms and tools, where possible.<br><br>Have you included the information requested as detailed in our Minimum Standards Reporting Checklist?	Yes
**Availability of data and materials**<br><br>All datasets and code on which the conclusions of the paper rely must be either included in your submission or deposited in publicly available repositories (where available and ethically appropriate), referencing such data using a unique identifier in the references and in the "Availability of Data and Materials" section of your manuscript.<br><br>Have you have met the above requirement as detailed in our Minimum Standards Reporting Checklist?	Yes
GigaScience has policies and guidelines in place for the use of generative AI-	Yes

writing tools such as ChatGPT. If you have used such writing tools to assist with writing the manuscript this must be declared and cited in the text. Authors should not list AI-writing tools and other AI-assisted technologies as an author or co-author and should acknowledge that they are fully responsible for text generated or refined by AI-writing tools.<p><br><br>A summary of use (particularly in the introduction or among methods) needs to be included at the end of the paper, and the outputs should also be included as a supplementary file hosted in GigaDB or other open repositories. Please read our guidelines for more information. <p><br><br>By submitting to GigaScience, you are aware of the journal's AI-writing tools policy, and if you have declared use of such tools below, you have acknowledged this where appropriate in your manuscript and have made a summary of use and outputs available. <p><br><b>AI-assisted writing tools have been used in the preparation of this manuscript?	

Powered by Editorial Manager® and ProduXion Manager® from Aries Systems Corporation

Manuscript

Click here to access/download;Manuscript;ChatGPT_Python_OtherLanguage

Translating short-form Python exercises to other programming languages using diverse prompting strategies

Stephen R. Piccolo[1], Harlan P. Stevens[1,2]

1 - Department of Biology, Brigham Young University, Provo, UT, USA

2 - Harvard Medical School, Harvard University, Boston, MA, USA

Please address correspondence to S.R.P. at stephen_piccolo@byu.edu.

Abstract

With the increasing complexity and quantity of data, life scientists rely on programming to automate analyses, enhance reproducibility, and facilitate collaboration. Scripting languages like Python are often favored for their simplicity and flexibility, enabling researchers to focus primarily on high-level tasks. Compiled languages such as C++ and Rust offer greater efficiency, making them preferable for intensive or repeated computations. In educational settings, instructors may wish to teach both types of languages and thus may wish to translate content from one programming language to another. In research contexts, researchers may wish to implement their ideas in one language before translating the code to another. However, translating between programming languages requires significant effort, prompting our interest in using large language models (LLMs) for semi-automated code translation. This study explores the use of an LLM (GPT-4) to translate 559 short-form programming exercises from Python into C++, Rust, Julia, and JavaScript. We used three prompting strategies—instructions only, code only, or both combined—and compared the translated code's output against the Python code's output. Translation success differed considerably by prompting strategy, and at least one strategy was successful for nearly

every exercise. The highest overall success rate occurred for Rust (99.5%), followed by JavaScript (98.9%), C++ (97.9%), and Julia (95.0%). Our findings demonstrate that LLMs can effectively translate small-scale programming exercises between languages, reducing the need for manual rewriting. To support education and research, we have manually translated all exercises that were not translated successfully through automation, and we have made our translations freely available.

Introduction

Due to the growing scale and complexity of biological data, computer-programming skills have become essential for modern life scientists[1]. Researchers use programming to automate tasks such as processing genomic datasets, running simulations, and creating visualizations. Writing code to carry out these tasks promotes reproducibility, enabling other scientists to scrutinize, validate, and extend computational workflows[2]. In turn, reproducibility fosters collaboration and accelerates scientific discovery.

When choosing a programming language, life scientists often consider both their own expertise and the demands of the task[3]. For high-level data analyses, scripting languages like Python and R are widely used[3–5]. These languages make it relatively easy to perform tasks such as importing and exporting data, performing statistical analyses, training machine-learning models, and generating graphics[6,7]. Because such analyses may be specific to a particular study and run only once, speed and resource efficiency are not always priorities. In contrast, tasks that are repeated many times, are computationally intensive, or require fine-grained control over memory often benefit from implementation in compiled languages like C++ or Rust[8]. For example, Burrows-Wheeler Aligner (used for aligning short DNA sequences)[9], Salmon (for transcript quantification)[10], and the Geospatial Data Abstraction Library (for geospatial data processing)[11] are all written primarily in compiled languages to maximize performance.

Scripting and compiled languages differ in their syntax, execution model, and levels of abstraction. In scripting languages, an interpreter executes code line by line. These languages typically feature relatively

simple syntax[12] and dynamic typing. They also abstract away complex operations such as memory management, garbage collection, and thread control. As a result, scientists can concentrate on high-level research tasks without needing to manage low-level system details. In contrast, most compiled languages are statically typed and require greater attention to system-level concerns like memory allocation and thread handling. While programming in compiled languages may be more time consuming and cognitively demanding, the resulting programs generally execute faster and use computing resources more efficiently[13,14].

In post-secondary educational settings, it is increasingly common for instructors to teach scripting rather than compiled languages in introductory programming courses[15–17]. By learning a scripting language at the outset, students might more easily master programming logic before moving to more advanced topics. However, evidence is mixed regarding whether starting with a scripting or compiled language better prepares students for more advanced programming tasks[18–21]. When students transition from scripting to compiled languages, they often struggle with new syntax, static typing, and manual memory management. To ease this transition, it may be pedagogically useful to have students reimplement familiar exercises—such as those completed previously in a scripting language—as they learn a compiled language. This practice might encourage comparisons between paradigms, reinforce core programming concepts, and reduce the cognitive load associated with learning lower-level programming techniques.

In research settings, scientists may wish to prioritize implementing and testing core logic before focusing on performance, resource usage, or security concerns[22]. This practice—commonly referred to as rapid prototyping—enables researchers to explore ideas and validate functionality quickly. One approach is to write the initial version of a program in a scripting language, where development is faster and more flexible. Once the logic is sound, the code can be translated into a compiled language to improve execution speed or scalability[23]. This workflow allows researchers to balance development efficiency with computational performance.

Proficiency in multiple programming languages enables researchers to translate code manually from one language to another. However, acquiring and maintaining fluency in multiple languages demands considerable time and energy—resources that could be spent on other research tasks. A promising alternative is to use large language models (LLMs), which can not only generate code from natural language prompts but also translate code between programming languages. If LLMs can perform such translations accurately and with minimal human input, they could facilitate rapid prototyping in research and support the development of educational materials that help learners transition between programming languages.

To date, most existing research in this area has focused on source-to-source translation[24], translation between compiled languages[25–28], and translation of entire projects[25,29]. Less attention has been paid to how these models could support transitions between scripting and compiled languages and on comparing prompting strategies.

We evaluated the ability of an LLM (ChatGPT 4) to translate short-form educational programming exercises from Python to four target languages: three compiled languages (C++, Rust, and Julia) and one scripting language (JavaScript). Our dataset consisted of 559 exercises originally written for Python; each includes both English-language instructions and code. Many of the exercises are oriented specifically toward the life sciences. To assess translation accuracy, we used unit-test results as a reference and performed manual reviews when necessary. We compared model performance across different input types and target languages and categorized error types. In addition to summarizing our findings, we introduce a novel educational resource containing validated solutions to all 559 exercises and all five programming languages. We anticipate that this resource will be useful for both education and research.

Materials and Methods

We gathered short-form, Python programming exercises from Austin et al.[30] (n = 426) and Piccolo et al. (n = 133)[31]. Each exercise came with instructions (directed at students and other learners), an example solution, and Python code to test students' solutions. We wrote Python code to generate spreadsheets that were structured consistently for the two sources. We reviewed each problem and adjusted the wording to improve clarity, make the instructions more consistent across the exercises, and use terminology that was less specific to Python programming. For example, we removed the term "Python" from the instructions and used "vector," "HashMap," and "null" instead of "list," "dictionary," and "None," respectively. We removed URLs from the instructions, corrected typographical errors, and altered the example solutions to improve clarity or succintness (in a few cases). We excluded exercises that were incompatible between languages. In particular, some of the Piccolo et al. exercises used the *pandas* and *seaborn* packages for analysis tasks[7,32], but corresponding packages were unavailable for the programming languages to which the exercises would be translated. In some cases, the example solutions from Piccolo et al. included data files to be used as inputs; we embedded paths to these files within the spreadsheets. When testing the generated code, we copied these files to the current working directory.

Using OpenAI's Chat Completions API, we evaluated the ability to translate the example solutions and test code for the Python exercises to other programming languages: C++, Rust, Julia, and Javascript. When invoking the API, we used version "gpt-4-0314" of the model and the default *temperature* setting of 0.7. For each exercise and programming language, we separately evaluated each of three input types: A) the instructions only, B) the Python example solution, or C) the instructions and the example solution. In all cases, we provided expected outputs with each prompt, after converting uppercase characters to lowercase.

When using only the instructions in the prompt, we provided the following guidance to the model (replacing "{otherlanguage}" with the programming-language name):

```
You are a helpful assistant who generates {otherlanguage} code. You
are given a prompt and some accompanying Python code for testing.
Implement {otherlanguage} code in response to the prompt. Translate
the testing code to {otherlanguage} and put it in a main() method. Do
not provide any comments on how the code works or *any other* text.
Provide *code only*. Surround all code with backticks.
```

When using only the Python code in the prompt, we provided the following guidance:

```
You are a helpful assistant who translates Python code to
{otherlanguage} code. The second part of the code is testing code.
Translate all of the code to {otherlanguage} and invoke it in a main()
method. Do not provide any comments on how the code works or *any
other* text. Provide *code only*. Surround all code with backticks.
```

When using the instructions and the Python code in the prompt, we provided the following guidance:

```
You are a helpful assistant who generates {otherlanguage} code. You
are given a prompt, some accompanying Python code for testing, and an
example in Python. Implement {otherlanguage} code in response to this
information. Translate the test code to {otherlanguage} and put it in
a main() method. Do not provide any comments on how the code works or
*any other* text. Provide *code only*. Surround all code with
backticks.
```

In all cases, after receiving translated code from the API, we attempted to compile the code (where applicable) and execute it locally. For C++, we used *Apple clang version 11.0.3 (clang-1103.0.32.59)*. For Rust, we used version 1.67.1 of *cargo*. For Julia, we used version 1.9.2. For Javascript, we used version

9.8.0 of *npm*. When the generated code required an additional package, we installed it using the relevant package manager.

To facilitate the evaluation process, we generated a spreadsheet for each prompting strategy and programming language. These spreadsheets contain the raw outputs generated by the model, the code parsed from these outputs, the standard output and standard error resulting from code execution, and whether the standard output (converted to lowercase) matched the expected output. In cases where the outputs did *not* match, we reviewed the outputs manually and recorded a high-level reason for the failure to match. In some cases, we deemed that even though the model's output did not perfectly match the expected output, the outputs were qualitatively identical. Many exercises require objects to be printed to standard output, yet conceptually identical objects may be represented differently when printed at runtime for different programming languages. For example, the Python interpreter typically displays strings using single quotes, whereas the Rust runtime displays strings with double quotes. Similarly, Python uses native syntax for floating-point numbers, while Rust prints such numbers with named fields. In our analysis, when a generated solution passed neither automated validation nor manual review, we repeated the process of generating and evaluating code—up to 10 times. The prompts were identical across these iterations, and no new information was provided to the models from previous failed attempts.

In cases where the LLM did not generate a passing solution for a given programming language after 10 attempts, we manually created a solution. After doing so, we executed the code and verified that its output matched the expected output (via automated or manual review).

Data and code availability

To perform these analyses, we wrote scripts for Python (version 3.9) and R (version 4.4.1)[33]. Additionally, we used *tidyverse* packages (version 2.0.0) and the *ComplexUpset* package (version 1.3.5) when analyzing the data[6,34]. These scripts are stored in an open-access repository at https://osf.io/4yxrf.

This repository also contains descriptions and code for the Python exercises, data files used as inputs for the exercises, and translated solutions for each language.

Results

We evaluated an LLM's ability to translate 559 short-form programming exercises from Python into four target languages: C++, Rust, Julia, and JavaScript. Each exercise was translated using one of three input types: (A) English-language instructions only (modified to avoid Python-specific terminology), (B) Python code only, or (C) both instructions and code. We considered a translation to be successful if it produced the expected result within 10 attempts.

Overall, translation was most successful for Rust and JavaScript (Table 1). The highest success rates—96.8% for Rust and 96.2% for JavaScript—were achieved when both instructions and code were provided. However, interestingly, C++ performed best (94.3%) when only the code was given, while Julia achieved its highest success rate (87.8%) with instructions alone.

We quantified performance levels when *at least one input type* led to a successful translation per exercise. For example, if the instructions-only prompt led to a successful translation but the code-only prompt did not, we counted this as a success. Using this approach, the model successfully translated 556 out of 559 exercises for Rust, yielding an overall success rate of 99.5%. By this measure, success rates reached at least 95.0% for all four programming languages (Table 1).

All target languages demonstrated performance improvements over successive iterations, with varying degrees of effectiveness, depending on the prompting strategy employed. The *Any* strategy—indicating at least one successful translation from any of the three input types—consistently outperformed individual prompting strategies across all programming languages (Figure 1). For Rust translations, performance improved rapidly in early iterations before beginning to plateau. Across all languages, there were diminishing returns, typically after approximately 7 iterations.

To provide insight on cross-language similarities and differences, we counted the number of times that translation was successful across *all* input types for each combination of target programming languages (Figure 2). Most commonly, translation was successful for all four languages. It was also common for three of the four languages—in different combinations—to be successful. For 22 of the exercises, translations were successful for none of the programming languages across all input types. Additionally, for each exercise and across all 4 target programming languages, we counted the number of times that a translation was successful for each combination of input types (Figure 3). When translations were *not* successful for all input types, the most common scenarios were for translations to be successful either for A) code only *and* both inputs or B) instructions only.

The sources of errors differed considerably across the programming languages. Most frequently, errors for the generated Rust and Julia code occurred at compile time or run time (Figure 4). However, for C++, logic errors were more common than compiling errors. For Javascript (a scripting language), logic errors were also the most common type. All four languages exhibited formatting mismatches—cases where the program output differed from the expected output in minor ways (e.g., spacing or punctuation); formatting errors were most common for the translated C++ code. In many instances, we deemed these differences acceptable upon manual review.

In cases where functional code had *not* been generated for *any* of the input types, we manually wrote functional code, using the generated code as a starting point. During this process, we used OpenAI's chatbot (ChatGPT 4) as an informal consultant. In each case, we tested the code using the same automated process that we used to validate the generated code. The amount of time it took to write functional code ranged from approximately one minute to three hours, depending on the exercise and programming language. The median time per exercise was considerably shorter for Javascript and Julia than for C++ and Rust (Figure 5).

To support future research and education efforts, we have made all of the source (Python) materials and translated code available for free, both as standalone files (see Methods) and via our CodeBuddy web

application (https://codebuddy.byu.edu). Via CodeBuddy, anyone with an Internet connection can create a free account and attempt to solve the exercises for any of the five programming languages.

Discussion

Researchers have long studied the ability to create statistical models of code structure[35–37] and use machines to translate code from one language to another[36,38,39]. In recent years, the field has shifted from rule-based transpilers to mostly machine-learning approaches, especially using neural networks[24,25,40]. Our work addresses the problem of translating short-form exercises consisting of relatively few (typically under 20) lines of code. Roziere et al. and others have facilitated work in this area by releasing examples of "parallel" solutions in multiple programming languages, making it feasible to train models on aligned examples of functionally equivalent code[24,41–43]. Additionally, researchers have emphasized the importance of using unit tests to verify that outputs are equivalent for different implementations of the same logic[43,44]. LLMs, supervised by humans, may be helpful in creating such tests[26,40]. Some researchers have sought to translate larger codebases; strategies have included translating one portion at a time independently[27] and using iterative-prompting strategies[45]. However, these attempts have been met with varied levels of success[26,27,29,45].

In this study, we have demonstrated that a general-purpose LLM is capable of facilitating semi-automated translation of short-form programming exercises from one programming language to another with relatively little human effort. Our research differs from prior work in multiple ways. Rather than using existing benchmark datasets that may have been used as training inputs for the GPT-4 model, we used exercises that were publicly available in Python but for which no translated versions were available (to our knowledge). Additionally, we asked the model to translate unit tests from Python to four target languages. Prior studies have focused primarily on Python, Java, and C++, although attention has shifted recently to Rust translations due to Rust's memory safety, particularly in systems programming

contexts[25–28]. Life scientists are turning to Rust as a way to improve the speed, safety, and reliability of their computational tools—particularly for tasks that are computationally and data intensive[8]. We included the Julia programming language in our analysis, in part because it combines the expressiveness of high-level languages like Python and R with execution speeds closer to those of C++ and Rust[22]. Of note, Julia is different from C++ and Rust in that it is just-in-time compiled and dynamically typed. Another difference between our work and others' is that we attempted three different prompting strategies and compared the LLM's ability to translate given these different inputs. Nearly all prior work has either used only instructions as prompts or attempted to translate from code in one programming language to another. Our results demonstrate that translation performance varies by prompting strategy and that these strategies are complementary. Finally, our work differs from prior work in that we 1) manually solved the exercises that were not automatically solved, 2) made these available for others to solve via a Web interface, and 3) have shared not only the source code and translated code—thus constituting a corpus of parallel examples for five languages—but also the full computational workflow we used to perform the analysis.

Whereas Python and Javascript are interpreted languages, three of the target languages (Rust, C++, and Julia) are compiled languages. Differences between programming paradigms provide some insight about whether translation successes and failures stem from language paradigm differences or from other factors in the translation process. Success rates for Rust and Javascript followed similar patterns to each other, suggesting that language paradigm alone does not account for translation performance. Instead, it points to the possibility that the availability and quality of training data for each language, as well as the model's exposure to how each language is commonly written and used in practice, play substantial roles.

Although it might be ideal to perform code translations in a fully automated manner, our analyses show that some manual review and translation are necessary for a large corpus of programming exercises. LLMs provide an opportunity to complement human efforts[46] and reduce the overall time and costs

involved. However, it is difficult to quantify these savings, which depend on factors like the labor cost of a human translator and the opportunity cost of redirecting humans' time away from other tasks.

In educational settings, semi-automatic translation may facilitate students' learning as they progress from one programming language to another. Similarly, it can help instructors adapt to industry trends and other factors that influence the choice of programming language in computational courses. Alternatively, an instructor or learner might wish to translate a compiled-language implementation of a particular algorithm to a scripting language so that its logic is more accessible.

In research settings, semi-automatic translation can enable rapid prototyping, in which logic is first implemented in a scripting language and later optimized for speed and other performance factors. In addition, this capability may take the place of creating interfaces between programming languages[47,48].

Our study is limited in several ways. The author (HPS) who manually solved the exercises that were not translated automatically was an undergraduate student. He had taken computer science and bioinformatics courses primarily in Python and C++. However, he was new to Rust and Julia and had minimal experience with JavaScript. Accordingly, the time required to manually solve these exercises may not reflect how long it would take a more experienced programmer to complete them—particularly one with deep familiarity across all target languages. Additionally, the LLM we used may perform differently in other contexts. While it showed promise for translating short, self-contained exercises, it may be considerably less effective for more complex tasks, such as translating large projects, interactive notebooks, or domain-specific libraries. Lastly, while our results broadly demonstrate the promise of LLMs for short-code translation, we only tested translation using GPT-4. While GPT-4 was state-of-the-art at the time of these experiments, newer or more specialized LLMs would likely produce different results.

Important work remains to identify best practices for using LLMs in translation settings. Potential solutions include integrating automated translation directly with human review, developing prompting

strategies that improve consistency across files, or training fine-tuned models on curated multi-language datasets. Additionally, interdisciplinary collaborations between domain experts and language model researchers may be essential to build robust, trustworthy tools for code translation in scientific and educational contexts.

Declarations

Ethics approval and consent to participate

Not applicable.

Consent for publication

Not applicable.

Competing interests

The authors declare no competing interests.

Financial disclosure

OpenAI, LLC provided free credits via their Researcher Access Program. We used these credits when accessing the Chat Completions API. No OpenAI employee had any involvement in the design of our study or in interpreting the results.

Author contributions

The following contributions are described using the CRediT Taxonomy[49].

SRP: Conceptualization, Formal Analysis, Investigation, Methodology, Project Administration, Resources, Software, Supervision, Visualization, Writing – Original Draft, Writing – Review & Editing

HPS: Investigation, Software, Writing – Review & Editing

AI-assisted writing

We used ChatGPT 4o to help with validating ideas and refining our writing during the writing process. We take full responsibility for the content of this paper.

Table

Table 1: Translation success rates after 10 iterations. For each programming language and input type, this table shows the success rate for translating to the target language within 10 iterations.

Target language	Instructions only	Code only	Instructions & code	Any
C++	89.6%	94.3%	92.1%	97.9%
Rust	**92.7%**	95.3%	**96.8%**	**99.5%**
Julia	87.8%	83.0%	85.2%	95.0%
Javascript	**92.7%**	**95.7%**	96.2%	98.9%

Figures

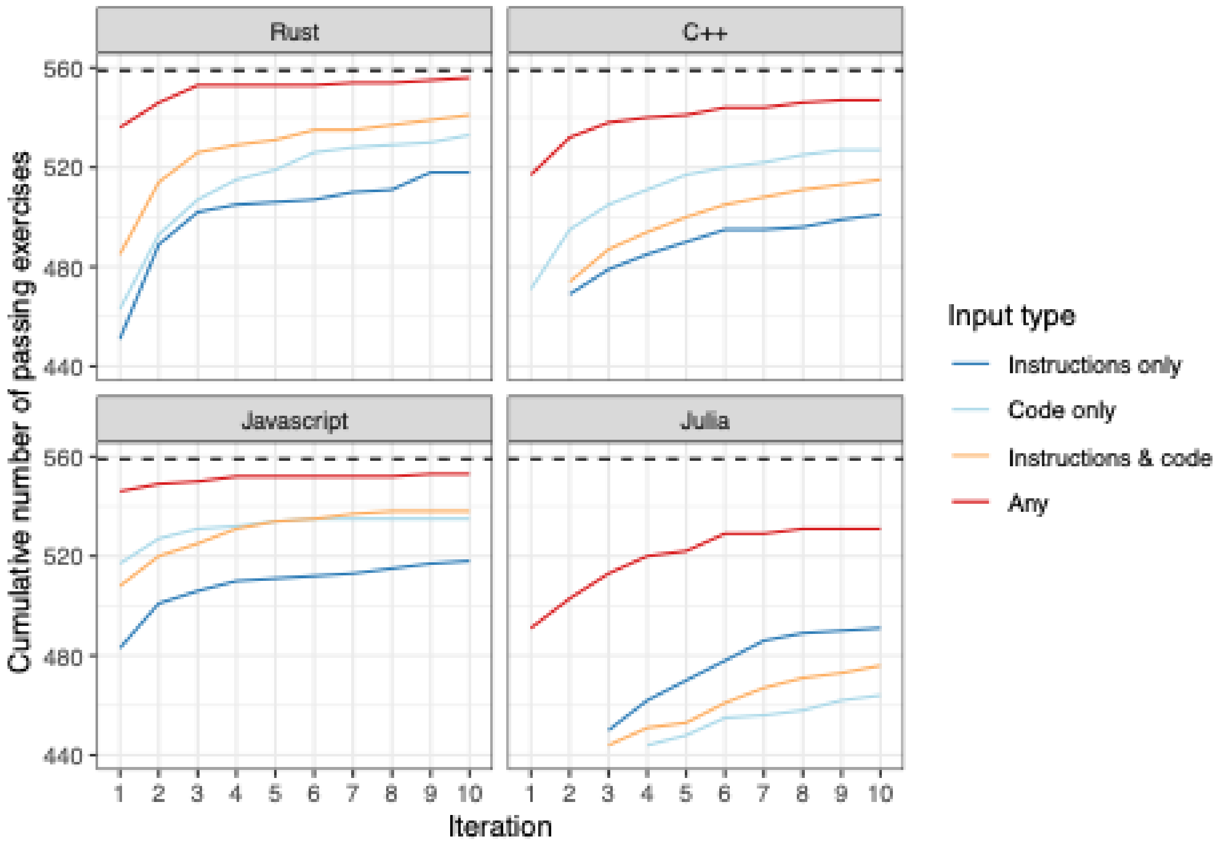

Figure 1: Cumulative number of passing exercises per iteration, input type, and programming language. When an exercise did not pass the tests on the first attempt, we repeated the code generation and validation process for up to ten attempts. This graph illustrates the cumulative number of exercises that passed the tests as the iterations progressed.

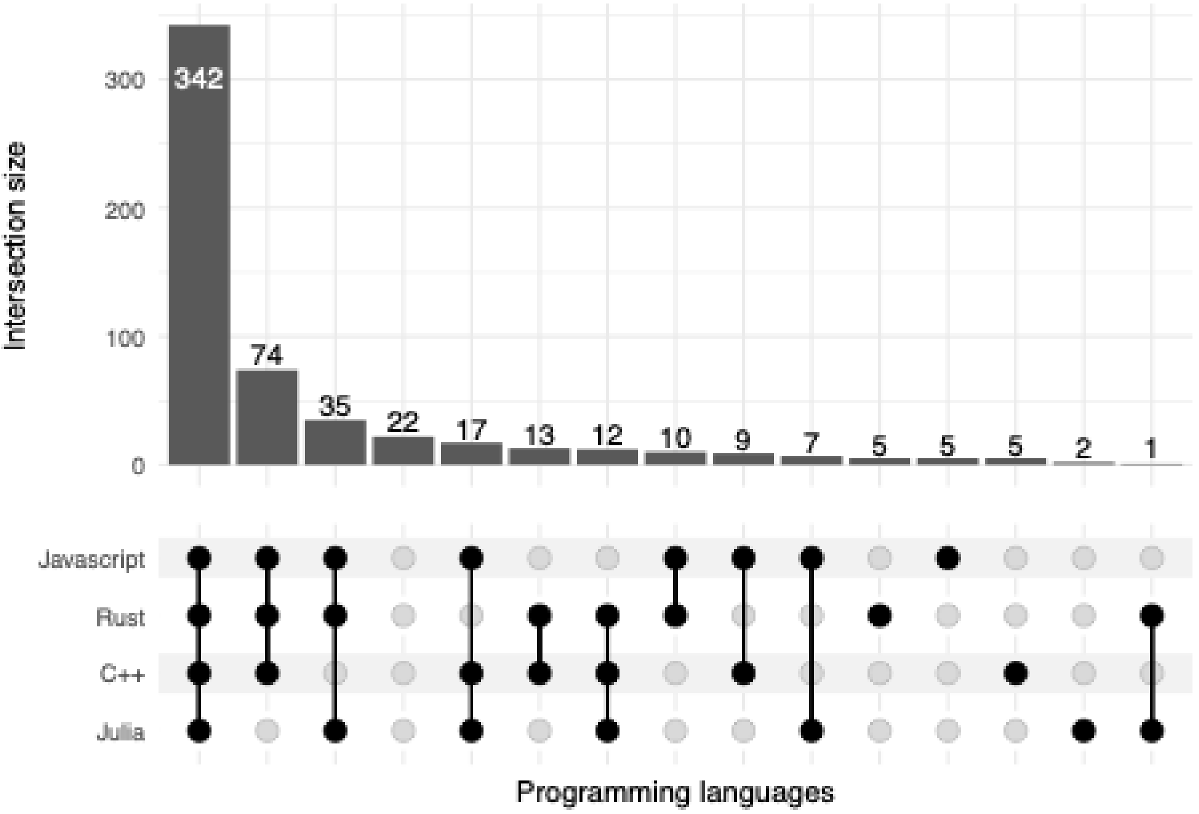

Figure 2: Overlap in translation success among programming languages. For each combination of target programming languages, we counted the number of times that translation was successful across all input types.

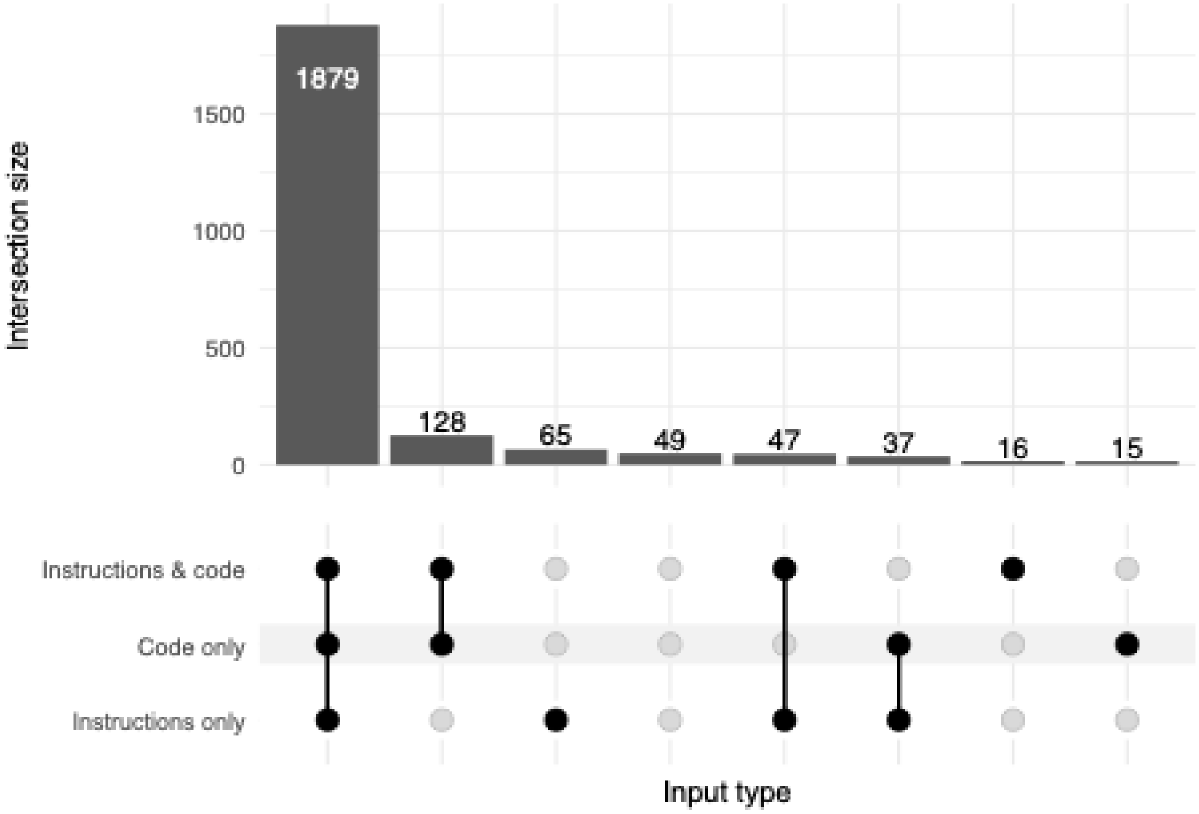

Figure 3: Overlap in translation success among input types. For each exercise and across all 4 target programming languages, we counted the number of times that a translation was successful for each combination of input types.

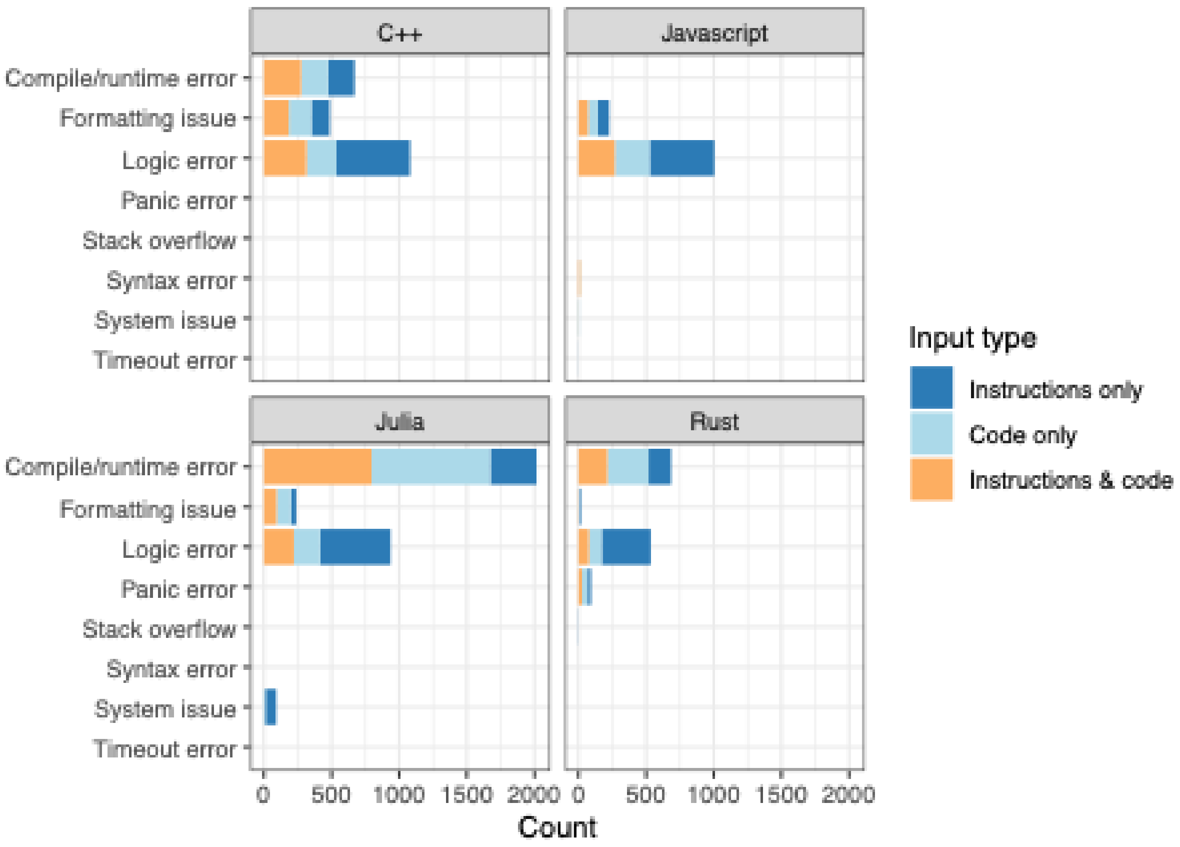

Figure 4: Outcome types for initially non-passing translated code. Upon compiling and/or executing translated code and finding that the code's output did not match the expected output for a given exercise, we categorized the reason for this mismatch. This chart summarizes these outcomes across the input types and programming languages.

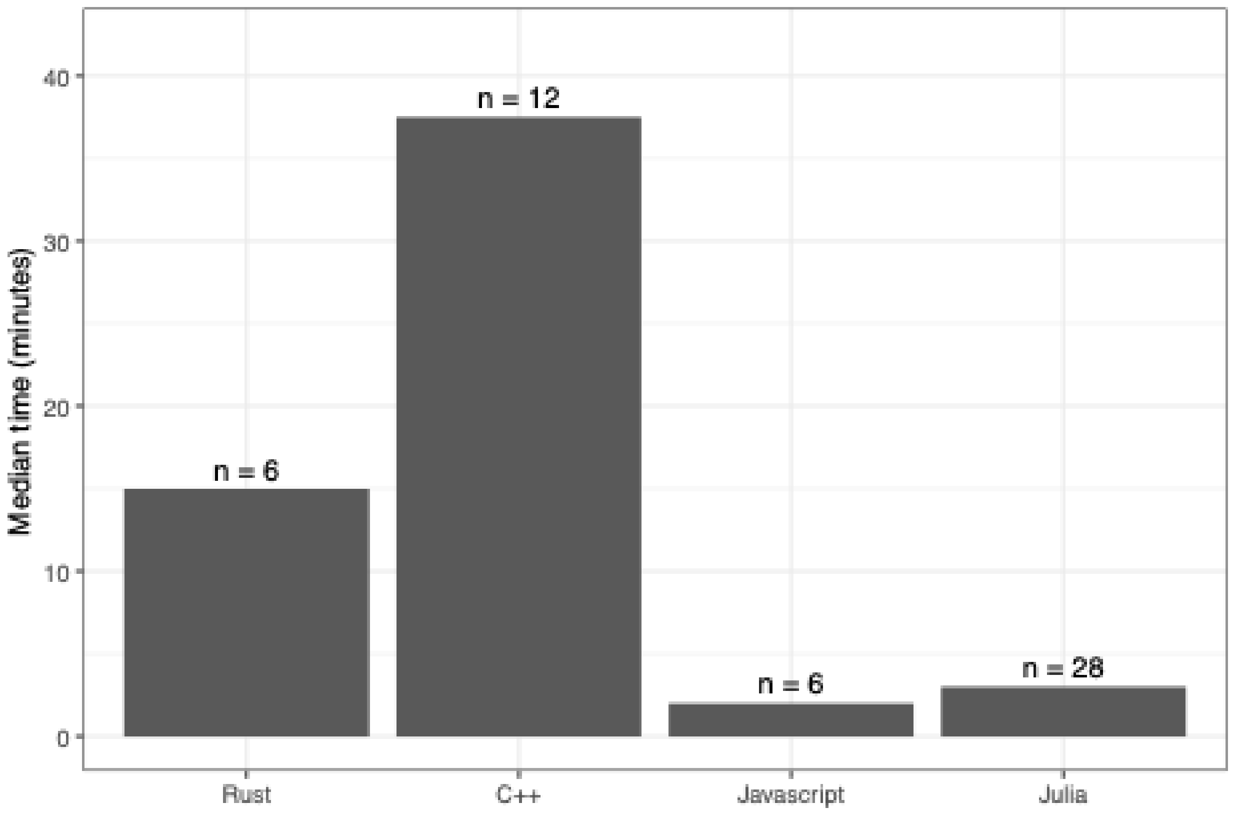

Figure 5: Approximate time to manually write code for exercises that were not successfully translated by the large language model. For exercises that were not successfully translated by the large language model for any of the prompting strategies, we manually created a functional solution. This graph illustrates the median time (in minutes) that we spent on creating solutions for each of the programming languages.

References

1. Barone, L., Williams, J. & Micklos, D. Unmet needs for analyzing biological big data: A survey of 704 NSF principal investigators. *PLOS Computational Biology* **13**, e1005755 (2017).

2. Piccolo, S. R. & Frampton, M. B. Tools and techniques for computational reproducibility. *Gigascience* **5**, 30 (2016).

3. Perkel, J. M. Which programming language should I use? A guide for early-career researchers. *Nature* **640**, 1116–1117 (2025).

4. Prabhu, P. *et al.* A survey of the practice of computational science. in *State of the Practice Reports* 1–12 (Association for Computing Machinery, New York, NY, USA, 2011). doi:10.1145/2063348.2063374.

5. Huber, W. *et al.* Orchestrating high-throughput genomic analysis with Bioconductor. *Nature Methods* **12**, 115–121 (2015).

6. Wickham, H. *et al.* Welcome to the tidyverse. *Journal of Open Source Software* **4**, 1686 (2019).

7. McKinney, W. Data Structures for Statistical Computing in Python. in *Proceedings of the 9th Python in Science Conference* 6 (2010).

8. JE, O. & CT, T. Why scientists are turning to Rust. *Nature* **588**, 185 (2020).

9. Li, H. & Durbin, R. Fast and accurate short read alignment with Burrows-Wheeler transform. *Bioinformatics (Oxford, England)* **25**, 1754–60 (2009).

10. Patro, R., Duggal, G., Love, M. I., Irizarry, R. A. & Kingsford, C. Salmon provides fast and bias-aware quantification of transcript expression. *Nature methods* **14**, 417–419 (2017).

11. GDAL/OGR contributors. *GDAL/OGR Geospatial Data Abstraction Software Library*. (Open Source Geospatial Foundation, 2024). doi:10.5281/zenodo.5884351.

12. Fourment, M. & Gillings, M. R. A comparison of common programming languages used in bioinformatics. *BMC Bioinformatics* **9**, 82 (2008).

13. Pereira, R. *et al.* Ranking programming languages by energy efficiency. *Science of Computer Programming* **205**, 102609 (2021).

14. Fourment, M. & Gillings, M. R. A comparison of common programming languages used in bioinformatics. *BMC Bioinformatics* **9**, 82 (2008).

15. Siegfried, R. M., Herbert-Berger, K. G., Leune, K. & Siegfried, J. P. Trends Of Commonly Used Programming Languages in CS1 And CS2 Learning. in *2021 16th International Conference on Computer Science & Education (ICCSE)* 407–412 (2021). doi:10.1109/ICCSE51940.2021.9569444.

16. Johnson, L. F. C in the first course considered harmful. *Commun. ACM* **38**, 99–101 (1995).

17. Mason, R. & Cooper, G. Introductory programming courses in Australia and New Zealand in 2013 - trends and reasons. in *Proceedings of the Sixteenth Australasian Computing Education Conference - Volume 148* 139–147 (Australian Computer Society, Inc., AUS, 2014).

18. Enbody, R. J., Punch, W. F. & McCullen, M. Python CS1 as preparation for C++ CS2. in *Proceedings of the 40th ACM technical symposium on Computer science education* 116–120 (Association for Computing Machinery, New York, NY, USA, 2009). doi:10.1145/1508865.1508907.

19. Alzahrani, N., Vahid, F., Edgcomb, A., Nguyen, K. & Lysecky, R. Python Versus C++: An Analysis of Student Struggle on Small Coding Exercises in Introductory Programming Courses. in *Proceedings of the 49th ACM Technical Symposium on Computer Science Education* 86–91 (Association for Computing Machinery, New York, NY, USA, 2018). doi:10.1145/3159450.3160586.

20. Balreira, D. G., Silveira, T. L. T. da & Wickboldt, J. A. Investigating the impact of adopting Python and C languages for introductory engineering programming courses. *Computer Applications in Engineering Education* **31**, 47–62 (2023).

21. Denny, P. *et al.* Novice Reflections During the Transition to a New Programming Language. in *Proceedings of the 53rd ACM Technical Symposium on Computer Science Education - Volume 1* vol. 1 948–954 (Association for Computing Machinery, New York, NY, USA, 2022).

22. Roesch, E. *et al.* Julia for biologists. *Nat Methods* **20**, 655–664 (2023).

23. Ekmekci, B., McAnany, C. E. & Mura, C. An Introduction to Programming for Bioscientists: A Python-Based Primer. *PLoS Comput Biol* **12**, e1004867 (2016).

24. Roziere, B., Lachaux, M.-A., Chanussot, L. & Lample, G. Unsupervised Translation of Programming Languages. in *Advances in Neural Information Processing Systems* vol. 33 20601–20611 (Curran Associates, Inc., 2020).

25. Eniser, H. F. *et al.* Towards Translating Real-World Code with LLMs: A Study of Translating to Rust. (2025) doi:10.48550/arXiv.2405.11514.

26. Nitin, V., Krishna, R. & Ray, B. SpecTra: Enhancing the Code Translation Ability of Language Models by Generating Multi-Modal Specifications. (2024) doi:10.48550/arXiv.2405.18574.

27. Shiraishi, M. & Shinagawa, T. Context-aware Code Segmentation for C-to-Rust Translation using Large Language Models. (2024) doi:10.48550/arXiv.2409.10506.

28. Hong, J. & Ryu, S. To Tag, or Not to Tag: Translating C's Unions to Rust's Tagged Unions. in *Proceedings of the 39th IEEE/ACM International Conference on Automated Software Engineering* 40–52 (Association for Computing Machinery, New York, NY, USA, 2024). doi:10.1145/3691620.3694985.

29. Zhang, H., David, C., Wang, M., Paulsen, B. & Kroening, D. Scalable, Validated Code Translation of Entire Projects using Large Language Models. (2024) doi:10.48550/arXiv.2412.08035.

30. Austin, J. *et al.* Program Synthesis with Large Language Models. (2021) doi:10.48550/arXiv.2108.07732.

31. Piccolo, S. R., Denny, P., Luxton-Reilly, A., Payne, S. H. & Ridge, P. G. Evaluating a large language model's ability to solve programming exercises from an introductory bioinformatics course. *PLOS Computational Biology* **19**, e1011511 (2023).

32. Waskom, M. L. Seaborn: Statistical data visualization. *Journal of Open Source Software* **6**, 3021 (2021).

33. R Core Team. *R: A Language and Environment for Statistical Computing*. (R Foundation for Statistical Computing, Vienna, Austria, 2024).

34. Krassowski, M., Arts, M., Lagger, C. & Max. Krassowski/complex-upset: V1.3.5. (2022) doi:10.5281/zenodo.7314197.

35. Hindle, A., Barr, E. T., Su, Z., Gabel, M. & Devanbu, P. On the naturalness of software. in *Proceedings of the 34th International Conference on Software Engineering* 837–847 (IEEE Press, Zurich, Switzerland, 2012).

36. Karaivanov, S., Raychev, V. & Vechev, M. Phrase-Based Statistical Translation of Programming Languages. in *Proceedings of the 2014 ACM International Symposium on New Ideas, New Paradigms,*

and Reflections on Programming & Software 173–184 (Association for Computing Machinery, New York, NY, USA, 2014). doi:10.1145/2661136.2661148.

37. Allamanis, M., Barr, E. T., Devanbu, P. & Sutton, C. A Survey of Machine Learning for Big Code and Naturalness. *ACM Comput. Surv.* **51**, 81:1–81:37 (2018).

38. Atkinson, R. *et al.* Experiences creating a portable cedar. *ACM SIGPLAN Notices* **24**, 322–329 (1989).

39. Yasumatsu, K. & Doi, N. SPiCE: A system for translating Smalltalk programs into a C environment. *IEEE Transactions on Software Engineering* **21**, 902–912 (1995).

40. Yang, Z. *et al.* Exploring and Unleashing the Power of Large Language Models in Automated Code Translation. *Proc. ACM Softw. Eng.* **1**, 71:1585–71:1608 (2024).

41. Puri, R. *et al.* CodeNet: A Large-Scale AI for Code Dataset for Learning a Diversity of Coding Tasks. *arXiv.org* (2021).

42. Ahmad, W. U., Tushar, M. G. R., Chakraborty, S. & Chang, K.-W. AVATAR: A Parallel Corpus for Java-Python Program Translation. in *Findings of the Association for Computational Linguistics: ACL 2023* (eds. Rogers, A., Boyd-Graber, J. & Okazaki, N.) 2268–2281 (Association for Computational Linguistics, Toronto, Canada, 2023). doi:10.18653/v1/2023.findings-acl.143.

43. Liu, J., Xia, C. S., Wang, Y. & Zhang, L. Is Your Code Generated by ChatGPT Really Correct? Rigorous Evaluation of Large Language Models for Code Generation. *arXiv.org* (2023).

44. Roziere, B. *et al.* Leveraging Automated Unit Tests for Unsupervised Code Translation. (2022) doi:10.48550/arXiv.2110.06773.

45. Pan, R. *et al.* Lost in Translation: A Study of Bugs Introduced by Large Language Models while Translating Code. in *Proceedings of the IEEE/ACM 46th International Conference on Software Engineering* 1–13 (Association for Computing Machinery, New York, NY, USA, 2024). doi:10.1145/3597503.3639226.

46. Weisz, J. D. *et al.* Perfection Not Required? Human-AI Partnerships in Code Translation. in *Proceedings of the 26th International Conference on Intelligent User Interfaces* 402–412 (Association for Computing Machinery, New York, NY, USA, 2021). doi:10.1145/3397481.3450656.

47. Extending Python with C or C++. *Python documentation*.

48. Eddelbuettel, D. & François, R. Rcpp: Seamless R and C++ integration. *Journal of statistical software* **40**, 1–18 (2011).

49. Brand, A., Allen, L., Altman, M., Hlava, M. & Scott, J. Beyond authorship: Attribution, contribution, collaboration, and credit. *Learned Publishing* **28**, 151–155 (2015).
