# Supplementary material for: Translating short-form Python exercises to other programming languages using diverse prompting strategies
Source: Gigascience. 2025 Dec 8;14:giaf149. doi: 10.1093/gigascience/giaf149 (PMC12758376; doi:10.1093/gigascience/giaf149)
Supplement: giaf149_GIGA-D-25-00265_Revision_1 [file giaf149_giga-d-25-00265_revision_1.pdf]

GigaScience

Translating short-form Python exercises to other programming languages using diverse prompting strategies

--Manuscript Draft--

**Manuscript Number:**	GIGA-D-25-00265R1
**Full Title:**	Translating short-form Python exercises to other programming languages using diverse prompting strategies
**Article Type:**	Research
**Funding Information:**	
**Abstract:**	With the increasing complexity and quantity of experimental and observational data, life scientists rely on programming to automate analyses, enhance reproducibility, and facilitate collaboration. Scripting languages like Python are often favored for their simplicity and flexibility, enabling researchers to focus primarily on high-level tasks. Compiled languages such as C++ and Rust offer greater efficiency, making them preferable for intensive or repeated computations. In educational settings, instructors may wish to teach both types of languages and thus may wish to translate content from one programming language to another. In research contexts, researchers may wish to implement their ideas in one language before translating the code to another. However, translating between programming languages requires significant effort, prompting our interest in using large language models (LLMs) for semi-automated code translation. This study explores the use of an LLM (GPT-4) to translate 559 short-form programming exercises from Python into C++, Rust, Julia, and JavaScript. We used three prompting strategies—instructions only, code only, or both combined—and compared the translated code's output against the Python code's output. Translation success differed considerably by prompting strategy, and at least one of the strategies tested was effective for nearly every exercise. The highest overall success rate occurred for Rust (99.5%), followed by JavaScript (98.9%), C++ (97.9%), and Julia (95.0%). Our findings demonstrate that LLMs can effectively translate small-scale programming exercises between languages, reducing the need for manual rewriting. To support education and research, we have manually translated all exercises that were not translated successfully through automation, and we have made our translations freely available.
**Corresponding Author:**	Stephen R Piccolo, Ph.D.<br>Brigham Young University<br>Provo, UT UNITED STATES
**Corresponding Author Secondary Information:**	
**Corresponding Author's Institution:**	Brigham Young University
**Corresponding Author's Secondary Institution:**	
**First Author:**	Stephen R Piccolo, Ph.D.
**First Author Secondary Information:**	
**Order of Authors:**	Stephen R Piccolo, Ph.D.
	Harlan P Stevens
**Order of Authors Secondary Information:**	
**Response to Reviewers:**	Thank you for taking time to review our manuscript entitled, "Translating short-form Python exercises to other programming languages using diverse prompting strategies." Below we provide a point-by-point response to the reviewers' comments.<br><br>We look forward to moving this manuscript forward to publication.<br><br>--

Powered by Editorial Manager® and ProduXion Manager® from Aries Systems Corporation

Dear Dr. Piccolo,

Your manuscript "Translating short-form Python exercises to other programming languages using diverse prompting strategies" (GIGA-D-25-00265) has been assessed by our reviewers. Based on these reports, and my own assessment as Editor, I am pleased to inform you that it is potentially acceptable for publication in GigaScience, once you have carried out some essential revisions suggested by our reviewers.

Their reports, together with any other comments, are below….

Best wishes,

Hongfang Zhang
GigaScience
www.gigasciencejournal.com

Please include a point-by-point within the 'Response to Reviewers' box in the submission system. Please ensure you describe additional experiments that were carried out and include a detailed rebuttal of any criticisms or requested revisions that you disagreed with. Please also ensure that your revised manuscript conforms to the journal style, which can be found in the Instructions for Authors on the journal homepage. If the data and code has been modified in the revision process please be sure to update the public versions of this too.

Reviewer reports:

Reviewer #1: In this work, Piccolo and Stevens investigate the effectiveness of different strategies for translating code snippets across multiple programming languages using a large language model (GPT-4). The work provides valuable insights into how various prompting approaches affect translation accuracy across different language pairs. The paper is well written, includes appropriate figures and tables, and is practically relevant to the growing field of AI-assisted code development. I recommend publication after only minor revisions.

> We thank the reviewer for taking the time to provide this review and for these positive comments.

The abstract states, "at least one strategy was successful for nearly every exercise." This phrasing may imply that there was a single strategy that was consistently effective, but I believe the authors mean to say that for each exercise, at least one of the strategies tested was effective. I recommend clarifying this ambiguity.

> Thank you for this suggestion. We have updated this part of the Abstract according to this suggestion.

The authors mention that the code snippets considered are generally 20 lines or less, but there is no analysis of how translation accuracy varies with code length within this range. It would be valuable to examine whether 5-line snippets are translated more accurately than 20-line snippets, for example. If such analysis is beyond the current scope, the authors could at least acknowledge this limitation and suggest it for future work.

> Thank you for this suggestion. We have added a figure and sentence to the results with an analysis of the relationship between Python code length and translation success. The relationship was statistically significant. (We used # of characters rather than # of lines as a surrogate for code complexity.)

Although the figures and tables provide a quantitative assessment of the results, the text itself often presents findings without specific metrics (e.g., percentages). This lack of specifics makes the text seem vague in places, even though the data is fully presented in the figures and tables. The authors shouldn't repeat all the figure/table values in the text, but mentioning specific metrics to highlight the most important findings might help make the text feel more precise. Here are specific instances where concrete percentages could replace vague language, to illustrate what I mean:

Powered by Editorial Manager® and ProduXion Manager® from Aries Systems Corporation

- "For Rust translations, performance improved rapidly in early iterations before beginning to plateau" (how much did it improve, perhaps in terms of percentage?)
- "Most commonly, translation was successful for all four languages" (how often exactly?)
- "It was also common for three of the four languages—in different combinations—to be successful" (how common, exactly?)
- "When translations were not successful for all input types, the most common scenarios were for translations to be successful either for A) code only and both inputs or B) instructions only" (how common?)

> Thank you. We have added these percentages in the places mentioned.

In summary, this manuscript presents valuable research on code translation strategies with practical implications for AI-assisted programming. The experimental design is appropriate, and the findings are interesting. I recommend only minor revisions.

> Thanks again!

Reviewer #2: This paper studies the effectiveness of ChatGPT4 in translating scripting code in Python into other languages. This is an increasingly common need: translating code across languages for education and research reproducibility.

The study works at a good scale—evaluating 559 exercises across four target languages.

The authors have shared data, scripts, and the CodeBuddy resource, which supports reproducibility. This is great!

Testing multiple prompting strategies (instructions only, code only, both) is a nice contribution. While this is common in NLP, it seems to be one of the first applications in this particular domain.

The writing is clear overall and the manuscript has a logical flow.

> We thank the reviewer for taking the time to provide this review and for these positive comments.

Major concern:

The authors manually reviewed failed tests and counted some outputs as "qualitatively identical" even if tests failed due to syntax or formatting issues. The criteria for what counts as "qualitatively identical" are quite subjective. Other papers have used automated equivalence checking both output and semantics to reduce this subjectivity. Maybe at least mention it as a limitation.

> Thank you. We now mention this as a limitation in the Discussion section.

Minor concerns:

Model Diversity: They only used GPT-4 and no other LLMs. While this is often expected in CS/NLP venues, it may be less of an issue here. The authors did discuss this as a limitation, so this is more of a minor point.

> Thank you. We agree that this is a limitation and acknowledge that future work could address this.

Data Leakage: The exercises come from public sources. Although the authors state that no translated versions existed to their knowledge, GPT-4 may have been exposed to them during training since it was trained on data on the Internet. Maybe they should mention this in their limitation.

> Thank you. We now address this limitation in the Discussion section.

	Prompt Transparency: The prompts are described in the text, but it's unclear if they are shown verbatim. Usually, it's helpful to provide exact prompts as a figure or in the supplementary materials to improve reproducibility.<br><br>> Thank you for this suggestion. We decided it would be best to provide the full prompt templates in the manuscript itself. As before, these templates have placeholders. Now they include both the user prompt and the system prompt for each scenario. We agree that this will support transparency.<br><br>Maybe use some more technical nlp terms? Right now their prompting strategy is called zero shot prompting, which is not mentioned at all.<br><br>> Thank you. Because the journal's audience is broad and includes many who are not NLP/ML experts, we preferred to use less technical language. However, we agree that the term zero-shot prompting, in particular, is relevant because it conveys the idea that the models can be effective even when not trained for the specific types of tasks we were completing. We have modified the Introduction to use this term and explain this concept.<br><br>There is no mention of the types of exercises used or a table of benchmark characteristics (e.g., lines of code, number of functions). Adding a taxonomy of exercise types (string manipulation, recursion, I/O, etc.) would help readers understand how representative the dataset is. It would also be valuable to include summary statistics (average code length, complexity) and compare these to success rates. This would give much clearer insight into how complexity affects translation success. Maybe at least add a summary table of taxonomy of exercise types.<br><br>> Thank you for this suggestion. We created definitions of exercise types and mapped each exercise to one of them. This is reported in the body of the manuscript and in a new figure. Additionally, we evaluated the relationship between code length (number of characters) as a simple surrogate for code complexity and found a statistically significant correlation. We also added a figure that addressed both of these topics.<br><br>For their retry strategy, the authors simply re-issued the same prompt up to 10 times without any additional feedback. It would be much more informative to experiment with richer feedback approaches like re-asking with the previous error message, or adding hints to see if these improve results. Maybe also mention it as a limitation.<br><br>> Thank you. We now address this as a limitation in the Discussion section.
**Additional Information:**	
**Question**	**Response**
Are you submitting this manuscript to a special series or article collection?	No
**Experimental design and statistics**<br><br>Full details of the experimental design and statistical methods used should be given in the Methods section, as detailed in our Minimum Standards Reporting Checklist. Information essential to interpreting the data presented should be made available in the figure legends.<br><br>Have you included all the information requested in your manuscript?	Yes

Powered by Editorial Manager® and ProduXion Manager® from Aries Systems Corporation

**Resources**<br><br>A description of all resources used, including antibodies, cell lines, animals and software tools, with enough information to allow them to be uniquely identified, should be included in the Methods section. Authors are strongly encouraged to cite Research Resource Identifiers (RRIDs) for antibodies, model organisms and tools, where possible.<br><br>Have you included the information requested as detailed in our Minimum Standards Reporting Checklist?	Yes
**Availability of data and materials**<br><br>All datasets and code on which the conclusions of the paper rely must be either included in your submission or deposited in publicly available repositories (where available and ethically appropriate), referencing such data using a unique identifier in the references and in the "Availability of Data and Materials" section of your manuscript.<br><br>Have you have met the above requirement as detailed in our Minimum Standards Reporting Checklist?	Yes
GigaScience has policies and guidelines in place for the use of generative AI-writing tools such as ChatGPT. If you have used such writing tools to assist with writing the manuscript this must be declared and cited in the text. Authors should not list AI-writing tools and other AI-assisted technologies as an author or co-author and should acknowledge that they are fully responsible for text generated or refined by AI-writing tools.<p><br><br>A summary of use (particularly in the introduction or among methods) needs to be included at the end of the paper, and the outputs should also be included as a supplementary file hosted in GigaDB or other open repositories. Please <a	Yes

href=https://academic.oup.com/gigascience/pages/editorial_policies_and_reporting_standards target="_new" > read our guidelines for more information. <p><br><br>By submitting to GigaScience, you are aware of the journal's AI-writing tools policy, and if you have declared use of such tools below, you have acknowledged this where appropriate in your manuscript and have made a summary of use and outputs available. <p><br><b>AI-assisted writing tools have been used in the preparation of this manuscript?	

Manuscript Click here to access/download;Manuscript;ChatGPT_Python_OtherLanguage

Translating short-form Python exercises to other programming languages using diverse prompting strategies

Stephen R. Piccolo[1], Harlan P. Stevens[1,2]

1 - Department of Biology, Brigham Young University, Provo, UT, USA

2 - Harvard Medical School, Harvard University, Boston, MA, USA

Please address correspondence to S.R.P. at stephen_piccolo@byu.edu.

Stephen R Piccolo [0000-0003-2001-5640]; Harlan P Stevens [0000-0003-1768-6777]

Abstract

With the increasing complexity and quantity of experimental and observational data, life scientists rely on programming to automate analyses, enhance reproducibility, and facilitate collaboration. Scripting languages like Python are often favored for their simplicity and flexibility, enabling researchers to focus primarily on high-level tasks. Compiled languages such as C++ and Rust offer greater efficiency, making them preferable for intensive or repeated computations. In educational settings, instructors may wish to teach both types of languages and thus may wish to translate content from one programming language to another. In research contexts, researchers may wish to implement their ideas in one language before translating the code to another. However, translating between programming languages requires significant effort, prompting our interest in using large language models (LLMs) for semi-automated code translation. This study explores the use of an LLM (GPT-4) to translate 559 short-form programming exercises from Python into C++, Rust, Julia, and JavaScript. We used three prompting strategies—instructions only, code only, or both combined—and compared the translated code's output against the

Python code's output. Translation success differed considerably by prompting strategy, and at least one of the strategies tested was effective for nearly every exercise. The highest overall success rate occurred for Rust (99.5%), followed by JavaScript (98.9%), C++ (97.9%), and Julia (95.0%). Our findings demonstrate that LLMs can effectively translate small-scale programming exercises between languages, reducing the need for manual rewriting. To support education and research, we have manually translated all exercises that were not translated successfully through automation, and we have made our translations freely available.

Introduction

Due to the growing scale and complexity of biological data, computer-programming skills have become essential for modern life scientists[1]. Researchers use programming to automate tasks such as processing genomic datasets, running simulations, and creating visualizations. Writing code to carry out these tasks promotes reproducibility, enabling other scientists to scrutinize, validate, and extend computational workflows[2]. In turn, reproducibility fosters collaboration and accelerates scientific discovery.

When choosing a programming language, life scientists often consider both their own expertise and the demands of the task[3]. For high-level data analyses, scripting languages like Python and R are widely used[3–5]. These languages make it relatively easy to perform tasks such as importing and exporting data, performing statistical analyses, training machine-learning models, and generating graphics[6,7]. Because such analyses may be specific to a particular study and run only once, speed and resource efficiency are not always priorities. In contrast, tasks that are repeated many times, are computationally intensive, or require fine-grained control over memory often benefit from implementation in compiled languages like C++ or Rust[8]. For example, Burrows-Wheeler Aligner (used for aligning short DNA sequences)[9], Salmon (for transcript quantification)[10], and the Geospatial Data Abstraction Library (for geospatial data processing)[11] are all written primarily in compiled languages to maximize performance.

Scripting and compiled languages differ in their syntax, execution model, and levels of abstraction. In scripting languages, an interpreter executes code line by line. These languages typically feature relatively simple syntax[12] and dynamic typing. They also abstract away complex operations such as memory management, garbage collection, and thread control. As a result, scientists can concentrate on high-level research tasks without needing to manage low-level system details. In contrast, most compiled languages are statically typed and require greater attention to system-level concerns like memory allocation and thread handling. While programming in compiled languages may be more time consuming and cognitively demanding, the resulting programs generally execute faster and use computing resources more efficiently[13,14].

In post-secondary educational settings, it is increasingly common for instructors to teach scripting rather than compiled languages in introductory programming courses[15–17]. By learning a scripting language at the outset, students might more easily master programming logic before moving to more advanced topics. However, evidence is mixed regarding whether starting with a scripting or compiled language better prepares students for more advanced programming tasks[18–21]. When students transition from scripting to compiled languages, they often struggle with new syntax, static typing, and manual memory management. To ease this transition, it may be pedagogically useful to have students reimplement familiar exercises—such as those completed previously in a scripting language—as they learn a compiled language. This practice might encourage comparisons between paradigms, reinforce core programming concepts, and reduce the cognitive load associated with learning lower-level programming techniques.

In research settings, scientists may wish to prioritize implementing and testing core logic before focusing on performance, resource usage, or security concerns[22]. This practice—commonly referred to as rapid prototyping—enables researchers to explore ideas and validate functionality quickly. One approach is to write the initial version of a program in a scripting language, where development is faster and more flexible. Once the logic is sound, the code can be translated into a compiled language to improve

execution speed or scalability[23]. This workflow allows researchers to balance development efficiency with computational performance.

Human proficiency in multiple programming languages enables researchers to translate code manually from one language to another. However, acquiring and maintaining fluency in multiple languages demands considerable time and energy—resources that could be spent on other research tasks. A promising alternative is to use large language models (LLMs), which can not only generate code from natural language prompts but also translate code between programming languages. Modern LLM chatbots typically support zero-shot prompting, which allows users to obtain meaningful outputs even when the underlying model has not encountered similar examples during training. If these models can perform code translations accurately and with minimal human input, they could accelerate rapid prototyping in research and aid in developing educational materials that help learners transition between programming languages.

To date, most existing research in this area has focused on source-to-source translation[24], translation between compiled languages[25–28], and translation of entire projects[25,29]. Less attention has been paid to how these models could support transitions between scripting and compiled languages and on comparing prompting strategies.

We evaluated the ability of an LLM (ChatGPT 4) to translate short-form educational programming exercises from Python to four target languages: three compiled languages (C++, Rust, and Julia) and one scripting language (JavaScript). Our dataset consisted of 559 exercises originally written for Python; each includes both English-language instructions and code. Many of the exercises are oriented specifically toward the life sciences. To assess translation accuracy, we used unit-test results as a reference and performed manual reviews when necessary. We compared model performance across different input types and target languages and categorized error types. In addition to summarizing our findings, we introduce a novel educational resource containing validated solutions to all 559 exercises and all five programming languages. We anticipate that this resource will be useful for both education and research.

Materials and Methods

We gathered short-form, Python programming exercises from Austin et al.[30] (n = 426) and Piccolo et al. (n = 133)[31]. Each exercise came with instructions (directed at students and other learners), an example solution, and Python code to test students' solutions. We wrote Python code to generate spreadsheets that were structured consistently for the two sources. We reviewed each problem and adjusted the wording to improve clarity, make the instructions more consistent across the exercises, and use terminology that was less specific to Python programming. For example, we removed the term "Python" from the instructions and used "vector," "HashMap," and "null" instead of "list," "dictionary," and "None," respectively. We removed URLs from the instructions, corrected typographical errors, and altered the example solutions to improve clarity or succintness (in a few cases). We excluded exercises that were incompatible between languages. In particular, some of the Piccolo et al. exercises used the *pandas* and *seaborn* packages for analysis tasks[7,32], but corresponding packages were unavailable for the programming languages to which the exercises would be translated. In some cases, the example solutions from Piccolo et al. included data files to be used as inputs; we embedded paths to these files within the spreadsheets. When testing the generated code, we copied these files to the current working directory.

For each exercise, we reviewed the Python code and student-directed instructions and assigned a category that reflected the types of programming skills necessary to solve the exercises. These categories and their descriptions are listed in Table 1.

Using OpenAI's Chat Completions API, we evaluated the ability to translate the example solutions and test code for the Python exercises to other programming languages: C++, Rust, Julia, and Javascript. When invoking the API, we used version "gpt-4-0314" of the model and the default *temperature* setting of 0.7. For each exercise and programming language, we separately evaluated each of three input types: A) the instructions only, B) the Python example solution, or C) the instructions and the example solution.

The API inputs consisted of the programming-task instructions and/or Python code (see Data Availability) and expected outputs.

When using only the instructions as the input, we provided the following user and systems messages to the API (with placeholders replaced with actual values for each exercise):

```
Prompt:

{instructions}

Python testing code (needs to be translated to {otherlanguage}):

{python_test_code}

Expected output (converted to lower case):

{expected_output}

You are a helpful assistant who generates {otherlanguage} code. You ar
e given a prompt and some accompanying Python code for testing. Implem
ent {otherlanguage} code in response to the prompt. Translate the test
ing code to {otherlanguage} and put it in a main() method. Do not prov
ide any comments on how the code works or *any other* text. Provide *c
ode only*. Surround all code with backticks.
```

When using only the Python code as the input, we provided the following user and systems messages to the API:

```
Python code to translate:

{python_code}

Python testing code (needs to be translated to {otherlanguage}):

{python_test_code}

Expected output (converted to lower case):

{expected_output}

You are a helpful assistant who translates Python code to {otherlanguage} code. The second part of the code is testing code. Translate all of the code to {otherlanguage} and invoke it in a main() method. Do not provide any comments on how the code works or *any other* text. Provide *code only*. Surround all code with backticks.
```

When using the instructions and the Python code in the prompt, we provided the following user and systems messages to the API:

```
Prompt:

{instructions}

Python testing code (needs to be translated to {otherlanguage}):

```

```
{python_test_code}

Expected output (converted to lower case):

{expected_output}

Example implementation of the code in Python:

{python_code}

You are a helpful assistant who generates {otherlanguage} code. You are given a prompt, some accompanying Python code for testing, and an example in Python. Implement {otherlanguage} code in response to this information. Translate the test code to {otherlanguage} and put it in a main() method. Do not provide any comments on how the code works or *any other* text. Provide *code only*. Surround all code with backticks.
```

In all cases, after receiving translated code from the API, we attempted to compile the code (where applicable) and execute it locally. For C++, we used *Apple clang version 11.0.3 (clang-1103.0.32.59)*. For Rust, we used version 1.67.1 of *cargo*. For Julia, we used version 1.9.2. For Javascript, we used version 9.8.0 of *npm*. When the generated code required an additional package, we installed it using the relevant package manager.

To facilitate the evaluation process, we generated a spreadsheet for each prompting strategy and programming language. These spreadsheets contain the raw outputs generated by the model, the code parsed from these outputs, the standard output and standard error resulting from code execution, and whether the standard output (converted to lowercase) matched the expected output. In cases where the

outputs did *not* match, we reviewed the outputs manually and recorded a high-level reason for the failure to match. In some cases, we deemed that even though the model's output did not perfectly match the expected output, the outputs were qualitatively identical. Many exercises require objects to be printed to standard output, yet conceptually identical objects may be represented differently when printed at runtime for different programming languages. For example, the Python interpreter typically displays strings using single quotation marks, whereas the Rust runtime displays strings with double quotation marks. Similarly, Python uses native syntax for floating-point numbers, while Rust prints such numbers with named fields. In our analysis, when a generated solution passed neither automated validation nor manual review, we repeated the process of generating and evaluating code—up to 10 times. The prompts were identical across these iterations, and no new information was provided to the models from previous failed attempts.

In cases where the LLM did not generate a passing solution for a given programming language after 10 attempts, we manually created a solution. After doing so, we executed the code and verified that its output matched the expected output (via automated or manual review).

To perform these analyses, we wrote scripts for Python (version 3.9) and R (version 4.4.1)[33]. Additionally, we used *tidyverse* packages (version 2.0.0) and the *ggupset* package (version 0.4.1) when analyzing the data[6,34].

Results

We evaluated an LLM's ability to translate 559 short-form programming exercises from Python into four target languages: C++, Rust, Julia, and JavaScript. Each exercise was translated using one of three input types: (A) English-language instructions only (modified to avoid Python-specific terminology), (B) Python code only, or (C) both instructions and code. We considered a translation to be successful if it produced the expected result within 10 attempts.

Overall, translation was most successful for Rust and JavaScript (Table 2). The highest success rates—96.8% for Rust and 96.2% for JavaScript—were achieved when both instructions and code were provided. However, interestingly, C++ performed best (94.3%) when only the code was given, while Julia achieved its highest success rate (87.8%) with instructions alone.

We quantified performance levels when *at least one input type* led to a successful translation per exercise. For example, if the instructions-only prompt led to a successful translation but the code-only prompt did not, we counted this as a success. Using this approach, the model successfully translated 556 out of 559 exercises for Rust, yielding an overall success rate of 99.5%. By this measure, success rates reached at least 95.0% for all four programming languages (Table 2).

All target languages demonstrated performance improvements over successive iterations, with varying degrees of effectiveness, depending on the prompting strategy employed. The *Any* strategy—indicating at least one successful translation from any of the three input types—consistently outperformed individual prompting strategies across all programming languages (Figure 1). As an example, for Rust translations, the proportion of passing exercises improved rapidly between iterations 1 and 4 —increasing by as much as 9.7%—before beginning to plateau. Proportions increased by a maximum of 3.2% between iterations 4 and 10 for Rust translations. Across all languages, there were often diminishing returns after approximately 7 iterations.

To provide insight on cross-language similarities and differences, we counted the number of times that translation was successful across *all* input types for each combination of target programming languages (Figure 2). Most commonly, translation was successful for all four languages (61.2% of scenarios). It was also common for three of the four languages—in different combinations—to be successful (24.7% of scenarios). For 22 (3.9%) of the exercises, translations were successful for none of the programming languages across all input types. Additionally, for each exercise and across all 4 target programming languages, we counted the number of times that a translation was successful for each combination of input types (Figure 3). Translations were successful for *all* three inputs types in 84.0% of scenarios. The

most common other scenarios were for translations to be successful either for A) code only *and* both inputs (5.7%) or B) instructions only (2.9%).

The sources of errors differed considerably across the programming languages. Most frequently, errors for the generated Rust and Julia code occurred at compile time or run time (Figure 4). However, for C++, logic errors were more common than compiling errors. For Javascript (a scripting language), logic errors were also the most common type. All four languages exhibited formatting mismatches—cases where the program output differed from the expected output in minor ways (e.g., spacing or punctuation); formatting errors were most common for the translated C++ code. In many instances, we deemed these differences acceptable upon manual review.

To provide additional insight into factors associated with successful translation, we manually assigned a category to each programming exercise (see Methods). Across all programming languages and input types, exercises in the “File Processing, Parsing & Domain-Specific Data Handling” (77.9%) and “Regular-Expression String Processing” (79.3%) categories were least often translated successfully. Exercises in the “String Processing (No Regex)” (95.9%) and “Arithmetic, Numeric Computation & Number Theory” (96.1%) categories were most often translated successfully. These success rates differed considerably across programming languages and input types (Figure 5). Using the number of characters in the Python solutions as an indicator of code complexity, we evaluated the relationship between code length and successful code translation. Across all programming languages and input types, there was a statistically signicant, negative correlation between code length and translation success (Spearman’s rho = -0.18, p = 2.4e-05; Figure 6). In simpler terms, translations were more successful for relatively short code examples.

In cases where functional code had *not* been generated for *any* of the input types, we manually wrote functional code, using the generated code as a starting point. During this process, we used OpenAI’s chatbot (ChatGPT 4) as an informal consultant. In each case, we tested the code using the same automated process that we used to validate the generated code. The amount of time it took to write

functional code ranged from approximately one minute to three hours, depending on the exercise and programming language. The median time per exercise was considerably shorter for Javascript and Julia than for C++ and Rust (Figure 7).

To support future research and education efforts, we have made all of the source (Python) materials and translated code available for free, both as standalone files (see Methods) and via our CodeBuddy web application (course name: "Sharpen Your Skills: Exercises in 5 Popular Programming Languages")[35]. Via CodeBuddy, anyone with an Internet connection can create a free account and attempt to solve the exercises for any of the five programming languages.

Discussion

Researchers have long studied the ability to create statistical models of code structure[36–38] and use machines to translate code from one language to another[37,39,40]. In recent years, the field has shifted from rule-based transpilers to mostly machine-learning approaches, especially using neural networks[24,25,41]. Our work addresses the problem of translating short-form exercises consisting of relatively few (typically under 20) lines of code. Roziere et al. and others have facilitated work in this area by releasing examples of "parallel" solutions in multiple programming languages, making it feasible to train models on aligned examples of functionally equivalent code[24,42–44]. Additionally, researchers have emphasized the importance of using unit tests to verify that outputs are equivalent for different implementations of the same logic[44,45]. LLMs, supervised by humans, may be helpful in creating such tests[26,41]. Some researchers have sought to translate larger codebases; strategies have included translating one portion at a time independently[27] and using iterative-prompting strategies[46]. However, these attempts have been met with varied levels of success[26,27,29,46].

In this study, we have demonstrated that a general-purpose LLM is capable of facilitating semi-automated translation of short-form programming exercises from one programming language to another with

relatively little human effort. Our research differs from prior work in multiple ways. Rather than using existing benchmark datasets that may have been used as training inputs for the GPT-4 model, we used exercises that were publicly available in Python. Additionally, we asked the model to translate unit tests from Python to four target languages. Prior studies have focused primarily on Python, Java, and C++, although attention has shifted recently to Rust translations due to Rust's memory safety, particularly in systems programming contexts[25–28]. Life scientists are turning to Rust as a way to improve the speed, safety, and reliability of their computational tools—particularly for tasks that are computationally and data intensive[8]. We included the Julia programming language in our analysis, in part because it combines the expressiveness of high-level languages like Python and R with execution speeds closer to those of C++ and Rust[22]. Of note, Julia is different from C++ and Rust in that it is just-in-time compiled and dynamically typed. Another difference between our work and others' is that we attempted three different prompting strategies and compared the LLM's ability to translate given these different inputs. Nearly all prior work has either used only instructions as prompts or attempted to translate from code in one programming language to another. Our results demonstrate that translation performance varies by prompting strategy and that these strategies are complementary. Finally, our work differs from prior work in that we 1) manually solved the exercises that were not automatically solved, 2) made these available for others to solve via a Web interface, and 3) have shared not only the source code and translated code—thus constituting a corpus of parallel examples for five languages—but also the full computational workflow we used to perform the analysis.

Whereas Python and Javascript are interpreted languages, three of the target languages (Rust, C++, and Julia) are compiled languages. Differences between programming paradigms provide some insight about whether translation successes and failures stem from language paradigm differences or from other factors in the translation process. Success rates for Rust and Javascript followed similar patterns to each other, suggesting that language paradigm alone does not account for translation performance. Instead, it points

to the possibility that the availability and quality of training data for each language, as well as the model's exposure to how each language is commonly written and used in practice, play substantial roles.

Although it might be ideal to perform code translations in a fully automated manner, our analyses show that some manual review and translation are necessary for a large corpus of programming exercises. LLMs provide an opportunity to complement human efforts[47] and reduce the overall time and costs involved. However, it is difficult to quantify these savings, which depend on factors like the labor cost of a human translator and the opportunity cost of redirecting humans' time away from other tasks.

In educational settings, semi-automatic translation may facilitate students' learning as they progress from one programming language to another. Similarly, it can help instructors adapt to industry trends and other factors that influence the choice of programming language in computational courses. Alternatively, an instructor or learner might wish to translate a compiled-language implementation of a particular algorithm to a scripting language so that its logic is more accessible.

In research settings, semi-automatic translation can enable rapid prototyping, in which logic is first implemented in a scripting language and later optimized for speed and other performance factors. In addition, this capability may take the place of creating interfaces between programming languages[48,49].

Our study is limited in several ways. The author (HPS) who manually solved the exercises that were not translated automatically was an undergraduate student. He had taken computer science and bioinformatics courses primarily in Python and C++. However, he was new to Rust and Julia and had minimal experience with JavaScript. Accordingly, the time required to manually solve these exercises may not reflect how long it would take a more experienced programmer to complete them—particularly a programmer with deep familiarity across all target languages. Additionally, the LLM we used may perform differently in other contexts. While it showed promise for translating short, self-contained exercises, it may be considerably less effective for more complex tasks, such as translating large projects, interactive notebooks, or domain-specific libraries. While our results broadly demonstrate the promise of

LLMs for short-code translation, we only tested translation using GPT-4. While GPT-4 was state-of-the-art at the time of these experiments, newer or more specialized LLMs would likely produce different results. Furthermore, to our knowledge, no translated versions of these datasets were publicly available at the time of our analysis. However, without specific knowledge of the sources used to train this proprietary model, it is impossible to confirm that the model had not previously been exposed to translated versions of the exercises. Another limitation is that we repeated the same prompt in each iteration. While this approach made retries more efficient, a more interactive process incorporating human feedback likely would have reduced the number of retries. One aspect of the process that did involve human interpretation was assessing whether code outputs were qualitatively identical between programming languages; developing automated equivalence checks (perhaps using an LLM) may have reduced the subjectivity of this process.

Important work remains to identify best practices for using LLMs in translation settings. Potential solutions include integrating automated translation directly with human review, developing prompting strategies that improve consistency across files, or training fine-tuned models on curated multi-language datasets. Additionally, interdisciplinary collaborations between domain experts and language model researchers may be essential to build robust, trustworthy tools for code translation in scientific and educational contexts.

Data Availability

An interactive version of the coding exercises can be found on the CodeBuddy website[50].

The scripts, programming task instructions, Python solution code, Python test code, and data files (where relevant) for the exercises, as well as translated solutions for each language are stored in an open-access repository at Zenodo[51].

Declarations

Ethics approval and consent to participate

Not applicable.

Consent for publication

Not applicable.

Competing interests

The authors declare no competing interests.

Financial disclosure

OpenAI, LLC provided free credits via their Researcher Access Program. We used these credits when accessing the Chat Completions API. No OpenAI employee had any involvement in the design of our study or in interpreting the results.

Author contributions

The following contributions are described using the CRediT Taxonomy[52].

SRP: Conceptualization, Formal Analysis, Investigation, Methodology, Project Administration, Resources, Software, Supervision, Visualization, Writing – Original Draft, Writing – Review & Editing

HPS: Investigation, Software, Writing – Review & Editing

Tables

Table 1: Exercise categories and descriptions. For each exercise, we reviewed the Python code and student-directed instructions and assigned a category that reflected the types of programming skills necessary to solve the exercises. These are listed in this table.

Term	Description
Basic Data Manipulation [DM]	Tasks involving creating, modifying, filtering, combining, or reorganizing standard data structures such as lists, vectors, tuples, sets, and dictionaries.
String Processing (No Regex) [SP]	Tasks that manipulate plain strings using basic operations such as splitting, joining, trimming, replacing characters, or checking prefixes/suffixes—without using regular expressions.
Regular-Expression String Processing [RX]	Tasks that require pattern matching, extraction, or substitution using explicit regular expressions.
Arithmetic, Numeric Computation & Number Theory [NC]	Tasks involving numerical calculations or properties of numbers, including sequences, digit manipulations, primes, divisors, summations, and other pure arithmetic logic.
Geometry, Scientific Formulas & Applied Math [GA]	Tasks using real-world mathematical formulas (e.g., area, volume, physics/biology formulas, unit conversions) where correctness depends on applying the proper equation.
Algorithms & Data Structures [AL]	Tasks requiring explicit algorithmic logic—such as searching, sorting, recursion, dynamic programming, subsequence/subarray algorithms, or other structured computational procedures.

Term	Description
Bitwise Operations, Binary Manipulation & Low-Level Logic [BW]	Tasks using binary arithmetic or bitwise operators (AND, OR, XOR, shifts) to examine or modify numerical values.
File Processing, Parsing & Domain-Specific Data Handling [FP]	Tasks that involve reading, writing, or transforming file contents, parsing external data formats, or performing computations based on domain-specific datasets (e.g., biological sequences, tabular data).

Table 2: Translation success rates after 10 iterations. For each programming language and input type, this table shows the success rate for translating to the target language within 10 iterations.

Target language	Instructions only	Code only	Instructions & code	Any
C++	89.6%	94.3%	92.1%	97.9%
Rust	**92.7%**	95.3%	**96.8%**	**99.5%**
Julia	87.8%	83.0%	85.2%	95.0%
Javascript	**92.7%**	**95.7%**	96.2%	98.9%

Figures

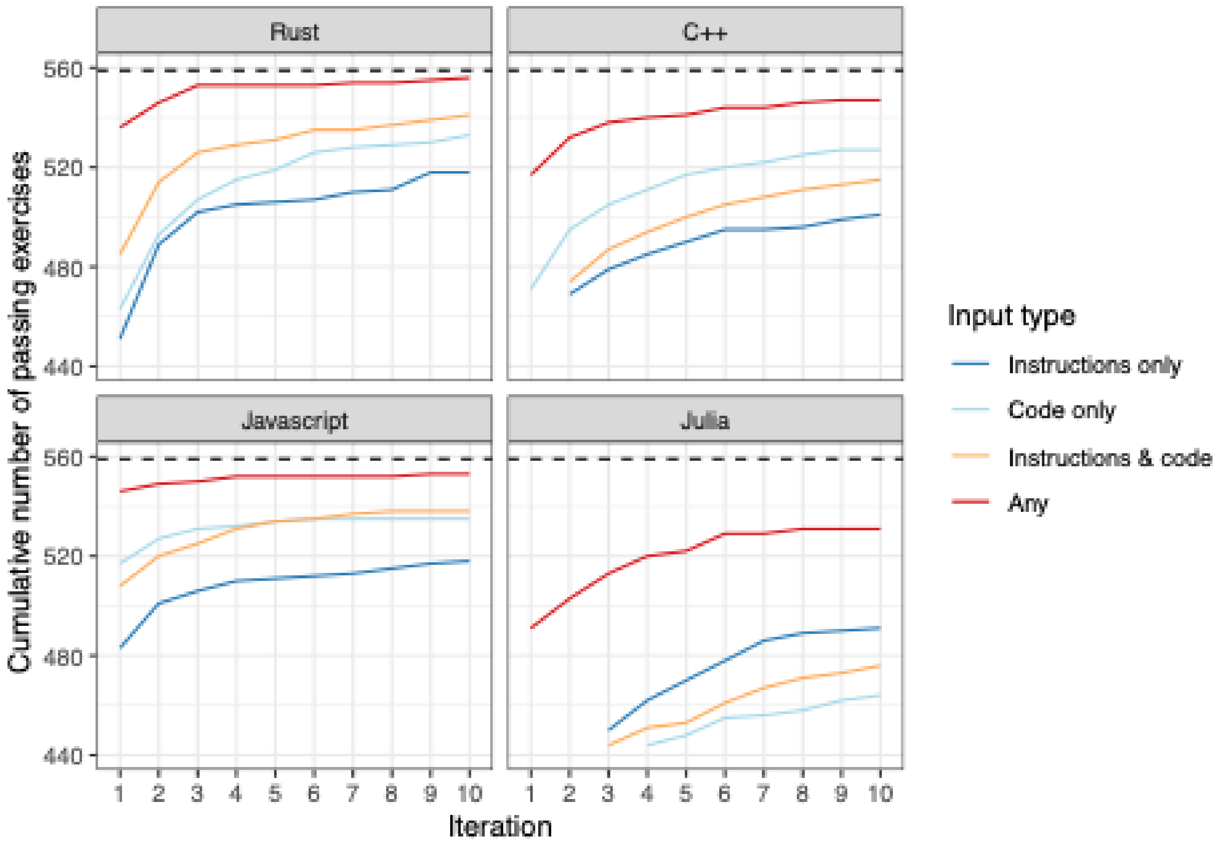

Figure 1: Cumulative number of passing exercises per iteration, input type, and programming language. When an exercise did not pass the tests on the first attempt, we repeated the code generation and validation process for up to ten attempts. This graph illustrates the cumulative number of exercises that passed the tests as the iterations progressed.

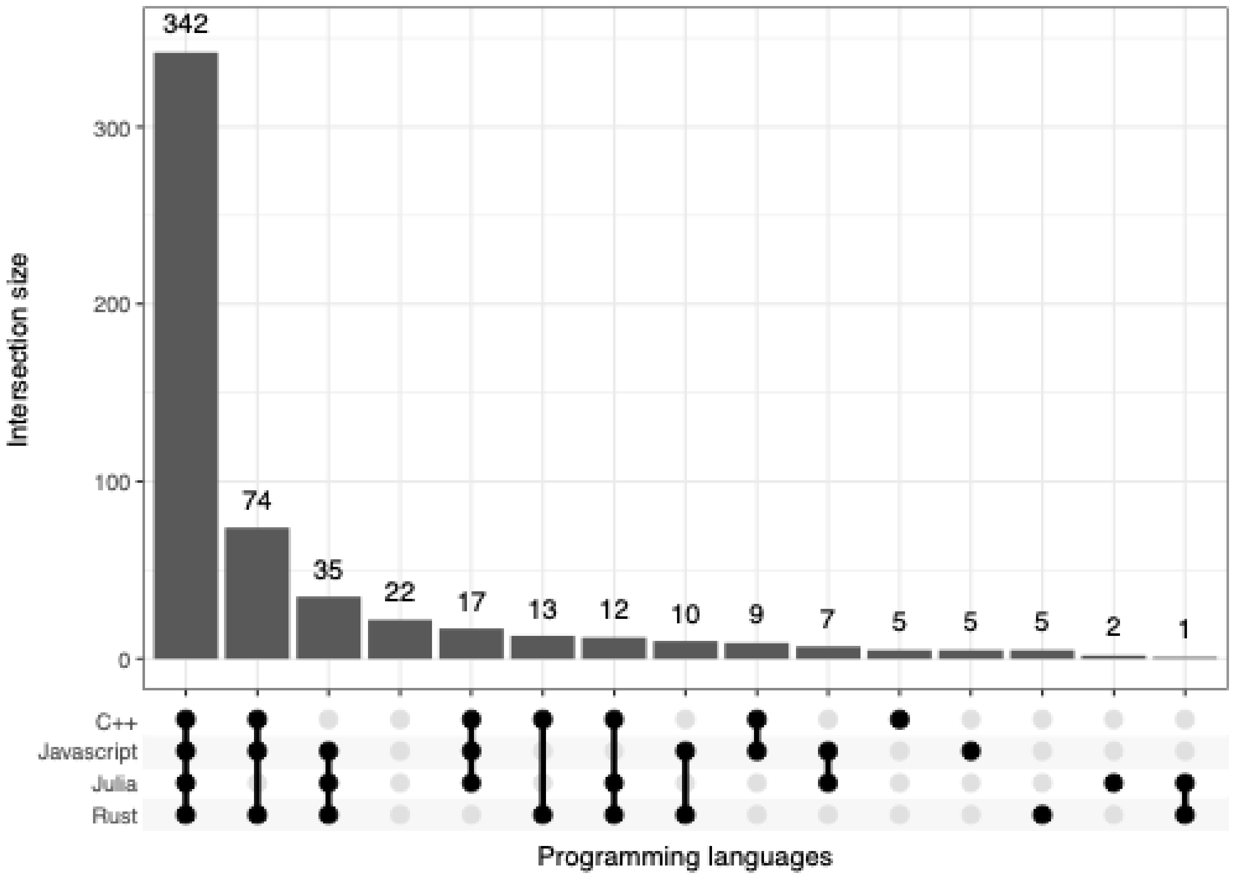

Figure 2: Overlap in translation success among programming languages. For each combination of target programming languages, we counted the number of times that translation was successful across all input types.

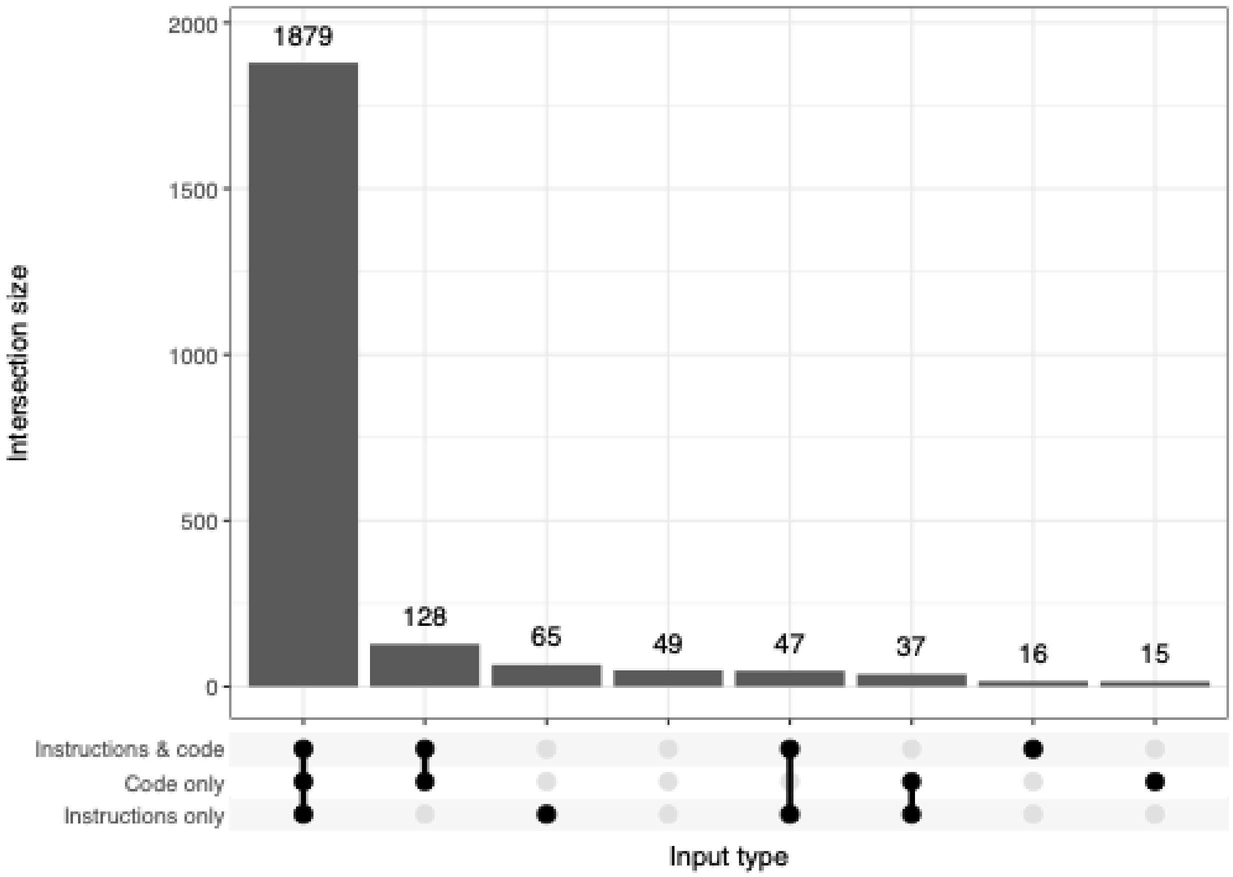

Figure 3: Overlap in translation success among input types. For each exercise and across all 4 target programming languages, we counted the number of times that a translation was successful for each combination of input types.

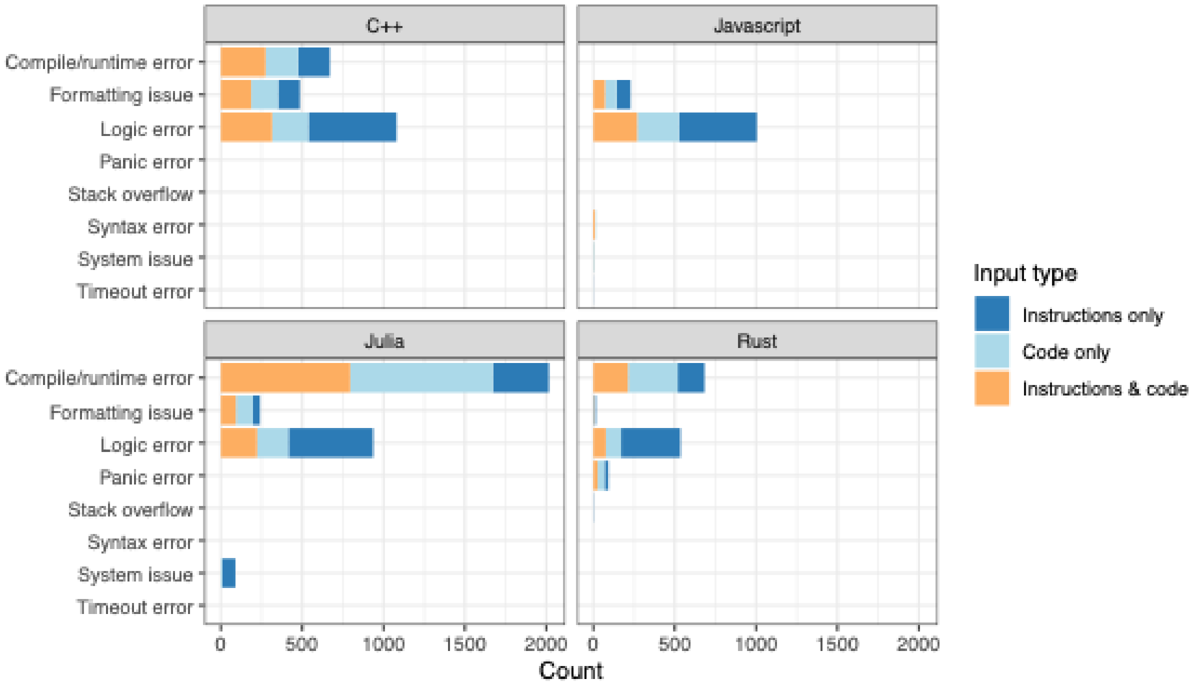

Figure 4: Outcome types for initially non-passing translated code. Upon compiling and/or executing translated code and finding that the code's output did not match the expected output for a given exercise, we categorized the reason for this mismatch. This chart summarizes these outcomes across the input types and programming languages.

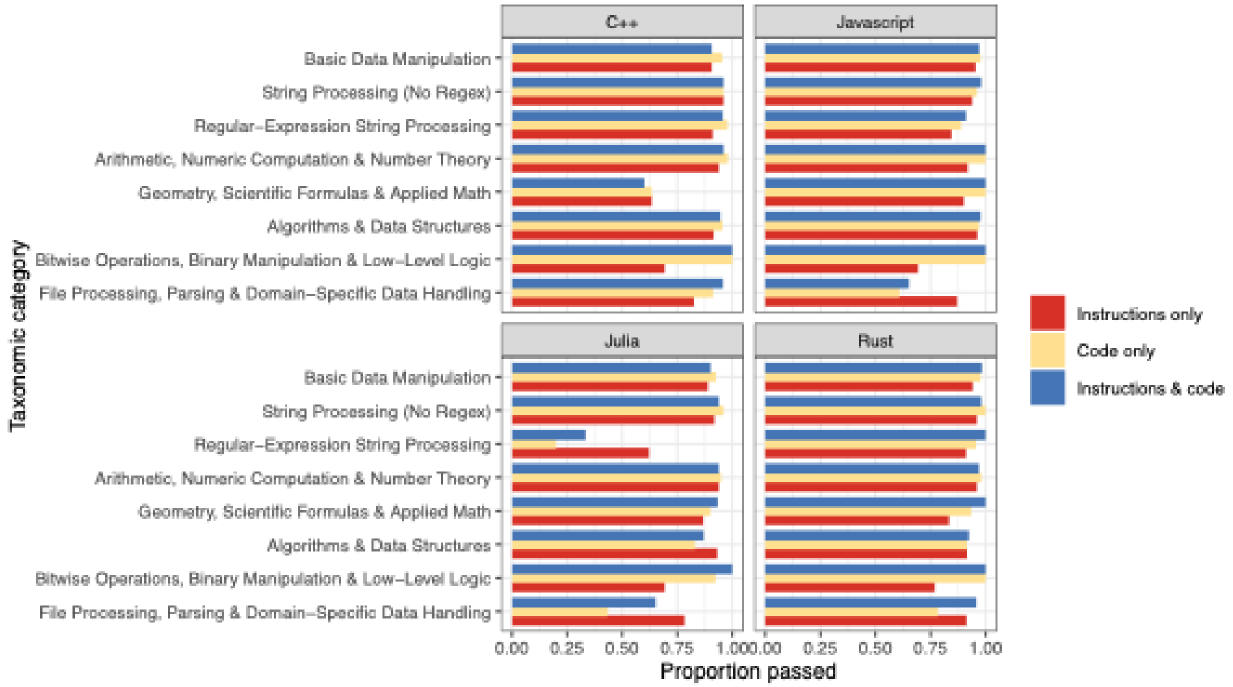

Figure 5: Proportion of exercises passed by taxonomic category. For each target programming language and input type, we calculated the proportion of exercises that were translated successfully per taxonomic category.

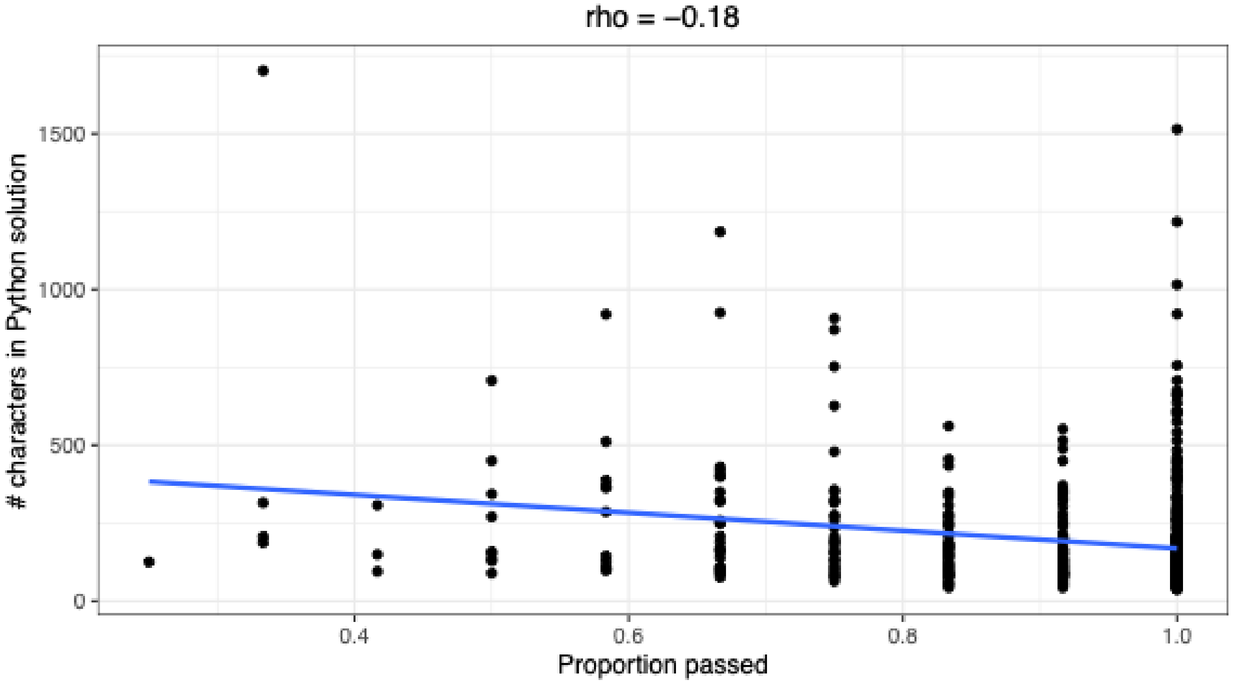

Figure 6: Relationship between code length and translation success. For each exercise, we calculated the number of characters in the Python solution. This plot illustrates the relationship between this measure of code length and the proportion of times that the exercise was translated successfully.

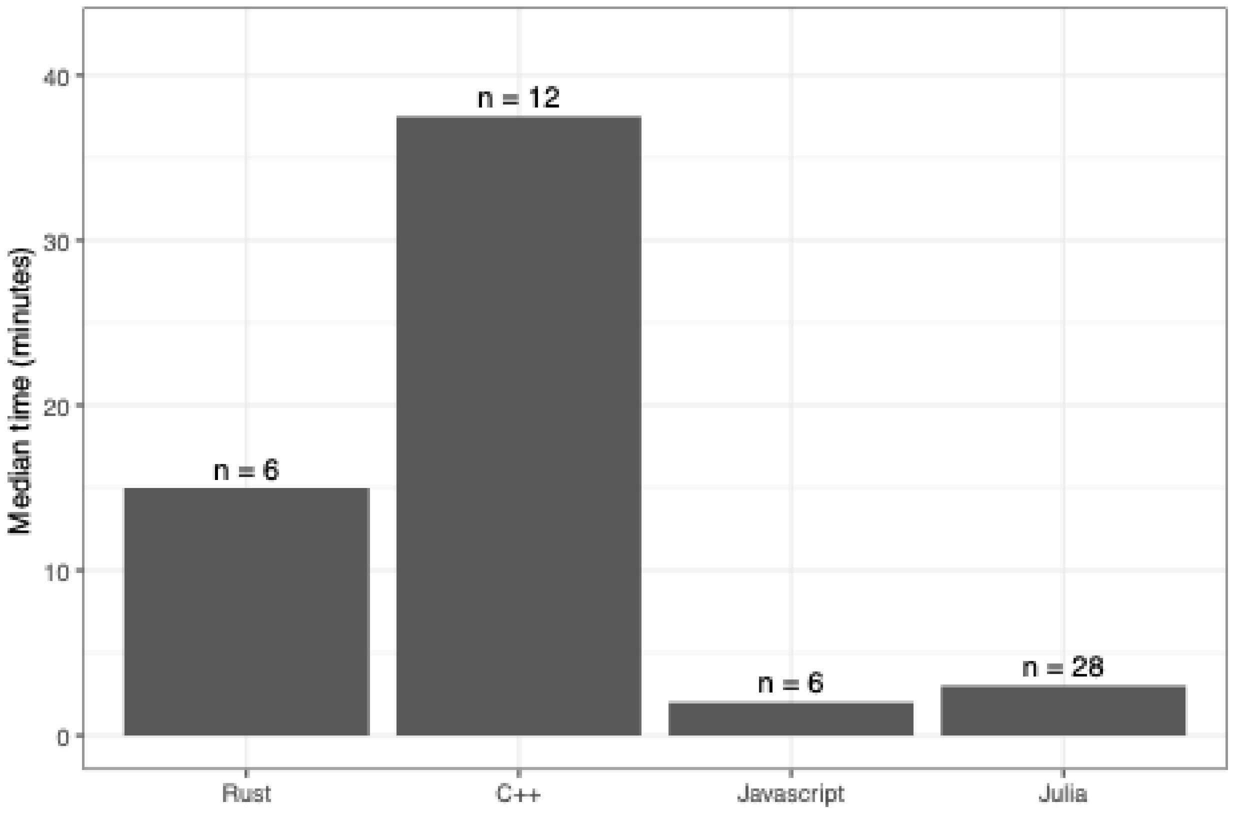

Figure 7: Approximate time to manually write code for exercises that were not successfully translated by the large language model. For exercises that were not successfully translated by the large language model for any of the prompting strategies, we manually created a functional solution. This graph illustrates the median time (in minutes) that we spent on creating solutions for each of the programming languages.

References

1. Barone, L., Williams, J. & Micklos, D. Unmet needs for analyzing biological big data: A survey of 704 NSF principal investigators. *PLOS Computational Biology* **13**, e1005755 (2017).
2. Piccolo, S. R. & Frampton, M. B. Tools and techniques for computational reproducibility. *GigaScience* **5**, 30 (2016).
3. Perkel, J. M. Which programming language should I use? A guide for early-career researchers. *Nature* **640**, 1116–1117 (2025).
4. Prabhu, P. *et al.* A survey of the practice of computational science. in *State of the Practice Reports* 1–12 (Association for Computing Machinery, New York, NY, USA, 2011). doi:10.1145/2063348.2063374.
5. Huber, W. *et al.* Orchestrating high-throughput genomic analysis with Bioconductor. *Nature Methods* **12**, 115–121 (2015).
6. Wickham, H. *et al.* Welcome to the tidyverse. *Journal of Open Source Software* **4**, 1686 (2019).
7. McKinney, W. Data Structures for Statistical Computing in Python. in *Proceedings of the 9th Python in Science Conference* 6 (2010).
8. JE, O. & CT, T. Why scientists are turning to Rust. *Nature* **588**, 185 (2020).
9. Li, H. & Durbin, R. Fast and accurate short read alignment with Burrows-Wheeler transform. *Bioinformatics (Oxford, England)* **25**, 1754–60 (2009).
10. Patro, R., Duggal, G., Love, M. I., Irizarry, R. A. & Kingsford, C. Salmon provides fast and bias-aware quantification of transcript expression. *Nature methods* **14**, 417–419 (2017).
11. GDAL/OGR contributors. *GDAL/OGR Geospatial Data Abstraction Software Library*. https://gdal.org (2024) doi:10.5281/zenodo.5884351.
12. Fourment, M. & Gillings, M. R. A comparison of common programming languages used in bioinformatics. *BMC Bioinformatics* **9**, 82 (2008).

13. Pereira, R. *et al.* Ranking programming languages by energy efficiency. *Science of Computer Programming* **205**, 102609 (2021).

14. Fourment, M. & Gillings, M. R. A comparison of common programming languages used in bioinformatics. *BMC Bioinformatics* **9**, 82 (2008).

15. Siegfried, R. M., Herbert-Berger, K. G., Leune, K. & Siegfried, J. P. Trends Of Commonly Used Programming Languages in CS1 And CS2 Learning. in *2021 16th International Conference on Computer Science & Education (ICCSE)* 407–412 (2021). doi:10.1109/ICCSE51940.2021.9569444.

16. Johnson, L. F. C in the first course considered harmful. *Commun. ACM* **38**, 99–101 (1995).

17. Mason, R. & Cooper, G. Introductory programming courses in Australia and New Zealand in 2013 - trends and reasons. in *Proceedings of the Sixteenth Australasian Computing Education Conference - Volume 148* 139–147 (Australian Computer Society, Inc., AUS, 2014).

18. Enbody, R. J., Punch, W. F. & McCullen, M. Python CS1 as preparation for C++ CS2. in *Proceedings of the 40th ACM technical symposium on Computer science education* 116–120 (Association for Computing Machinery, New York, NY, USA, 2009). doi:10.1145/1508865.1508907.

19. Alzahrani, N., Vahid, F., Edgcomb, A., Nguyen, K. & Lysecky, R. Python Versus C++: An Analysis of Student Struggle on Small Coding Exercises in Introductory Programming Courses. in *Proceedings of the 49th ACM Technical Symposium on Computer Science Education* 86–91 (Association for Computing Machinery, New York, NY, USA, 2018). doi:10.1145/3159450.3160586.

20. Balreira, D. G., Silveira, T. L. T. da & Wickboldt, J. A. Investigating the impact of adopting Python and C languages for introductory engineering programming courses. *Computer Applications in Engineering Education* **31**, 47–62 (2023).

21. Denny, P. *et al.* Novice Reflections During the Transition to a New Programming Language. in *Proceedings of the 53rd ACM Technical Symposium on Computer Science Education - Volume 1* vol. 1 948–954 (Association for Computing Machinery, New York, NY, USA, 2022).

22. Roesch, E. *et al.* Julia for biologists. *Nature Methods* **20**, 655–664 (2023).

23. Ekmekci, B., McAnany, C. E. & Mura, C. An Introduction to Programming for Bioscientists: A Python-Based Primer. *PLoS Computational Biology* **12**, e1004867 (2016).

24. Roziere, B., Lachaux, M.-A., Chanussot, L. & Lample, G. Unsupervised Translation of Programming Languages. in *Advances in Neural Information Processing Systems* vol. 33 20601–20611 (Curran Associates, Inc., 2020).

25. Eniser, H. F. *et al.* Towards Translating Real-World Code with LLMs: A Study of Translating to Rust. (2025) doi:10.48550/arXiv.2405.11514.

26. Nitin, V., Krishna, R. & Ray, B. SpecTra: Enhancing the Code Translation Ability of Language Models by Generating Multi-Modal Specifications. (2024) doi:10.48550/arXiv.2405.18574.

27. Shiraishi, M. & Shinagawa, T. Context-aware Code Segmentation for C-to-Rust Translation using Large Language Models. (2024) doi:10.48550/arXiv.2409.10506.

28. Hong, J. & Ryu, S. To Tag, or Not to Tag: Translating C's Unions to Rust's Tagged Unions. in *Proceedings of the 39th IEEE/ACM International Conference on Automated Software Engineering* 40–52 (Association for Computing Machinery, New York, NY, USA, 2024). doi:10.1145/3691620.3694985.

29. Zhang, H., David, C., Wang, M., Paulsen, B. & Kroening, D. Scalable, Validated Code Translation of Entire Projects using Large Language Models. (2024) doi:10.48550/arXiv.2412.08035.

30. Austin, J. *et al.* Program Synthesis with Large Language Models. (2021) doi:10.48550/arXiv.2108.07732.

31. Piccolo, S. R., Denny, P., Luxton-Reilly, A., Payne, S. H. & Ridge, P. G. Evaluating a large language model's ability to solve programming exercises from an introductory bioinformatics course. *PLOS Computational Biology* **19**, e1011511 (2023).

32. Waskom, M. L. Seaborn: Statistical data visualization. *Journal of Open Source Software* **6**, 3021 (2021).

33. R Core Team. *R: A Language and Environment for Statistical Computing*. https://www.R-project.org/ (2024).

34. Ahlmann-Eltze, C. *Ggupset: Combination Matrix Axis for 'Ggplot2' to Create 'UpSet' Plots*. https://CRAN.R-project.org/package=ggupset (2025) doi:10.32614/CRAN.package.ggupset.

35. Piccolo, S. R., Tuft, E., Tatlow, P. J., Eliason, Z. & Stephenson, A. CodeBuddy: A Programming Assignment Management System for Short-Form Exercises Journal of Open Research Software. (2025) doi:10.5334/jors.503.

36. Hindle, A., Barr, E. T., Su, Z., Gabel, M. & Devanbu, P. On the naturalness of software. in *Proceedings of the 34th International Conference on Software Engineering* 837–847 (IEEE Press, Zurich, Switzerland, 2012).

37. Karaivanov, S., Raychev, V. & Vechev, M. Phrase-Based Statistical Translation of Programming Languages. in *Proceedings of the 2014 ACM International Symposium on New Ideas, New Paradigms, and Reflections on Programming & Software* 173–184 (Association for Computing Machinery, New York, NY, USA, 2014). doi:10.1145/2661136.2661148.

38. Allamanis, M., Barr, E. T., Devanbu, P. & Sutton, C. A Survey of Machine Learning for Big Code and Naturalness. *ACM Comput. Surv.* **51**, 81:1–81:37 (2018).

39. Atkinson, R. *et al.* Experiences creating a portable cedar. *ACM SIGPLAN Notices* **24**, 322–329 (1989).

40. Yasumatsu, K. & Doi, N. SPiCE: A system for translating Smalltalk programs into a C environment. *IEEE Transactions on Software Engineering* **21**, 902–912 (1995).

41. Yang, Z. *et al.* Exploring and Unleashing the Power of Large Language Models in Automated Code Translation. *Proc. ACM Softw. Eng.* **1**, 71:1585–71:1608 (2024).

42. Puri, R. *et al.* CodeNet: A Large-Scale AI for Code Dataset for Learning a Diversity of Coding Tasks. *arXiv.org* (2021).

43. Ahmad, W. U., Tushar, M. G. R., Chakraborty, S. & Chang, K.-W. AVATAR: A Parallel Corpus for Java-Python Program Translation. in *Findings of the Association for Computational Linguistics: ACL 2023* (eds. Rogers, A., Boyd-Graber, J. & Okazaki, N.) 2268–2281 (Association for Computational Linguistics, Toronto, Canada, 2023). doi:10.18653/v1/2023.findings-acl.143.

44. Liu, J., Xia, C. S., Wang, Y. & Zhang, L. Is Your Code Generated by ChatGPT Really Correct? Rigorous Evaluation of Large Language Models for Code Generation. *arXiv.org* (2023).

45. Roziere, B. *et al.* Leveraging Automated Unit Tests for Unsupervised Code Translation. (2022) doi:10.48550/arXiv.2110.06773.

46. Pan, R. *et al.* Lost in Translation: A Study of Bugs Introduced by Large Language Models while Translating Code. in *Proceedings of the IEEE/ACM 46th International Conference on Software Engineering* 1–13 (Association for Computing Machinery, New York, NY, USA, 2024). doi:10.1145/3597503.3639226.

47. Weisz, J. D. *et al.* Perfection Not Required? Human-AI Partnerships in Code Translation. in *Proceedings of the 26th International Conference on Intelligent User Interfaces* 402–412 (Association for Computing Machinery, New York, NY, USA, 2021). doi:10.1145/3397481.3450656.

48. Extending Python with C or C++. *Python documentation*.

49. Eddelbuettel, D. & François, R. Rcpp: Seamless R and C++ integration. *Journal of statistical software* **40**, 1–18 (2011).

50. CodeBuddy website: https://codebuddy.byu.edu.

51. Piccolo SR, Stevens HP. Supplementary files for "Translating short-form Python exercises to other programming languages using diverse prompting strategies." Zenodo. 2025. https://doi.org/10.5281/zenodo.17723732

52. Brand, A., Allen, L., Altman, M., Hlava, M. & Scott, J. Beyond authorship: Attribution, contribution, collaboration, and credit. *Learned Publishing* **28**, 151–155 (2015).

Figure 1

Click here to access/download;Figure;Figure_1.pdf

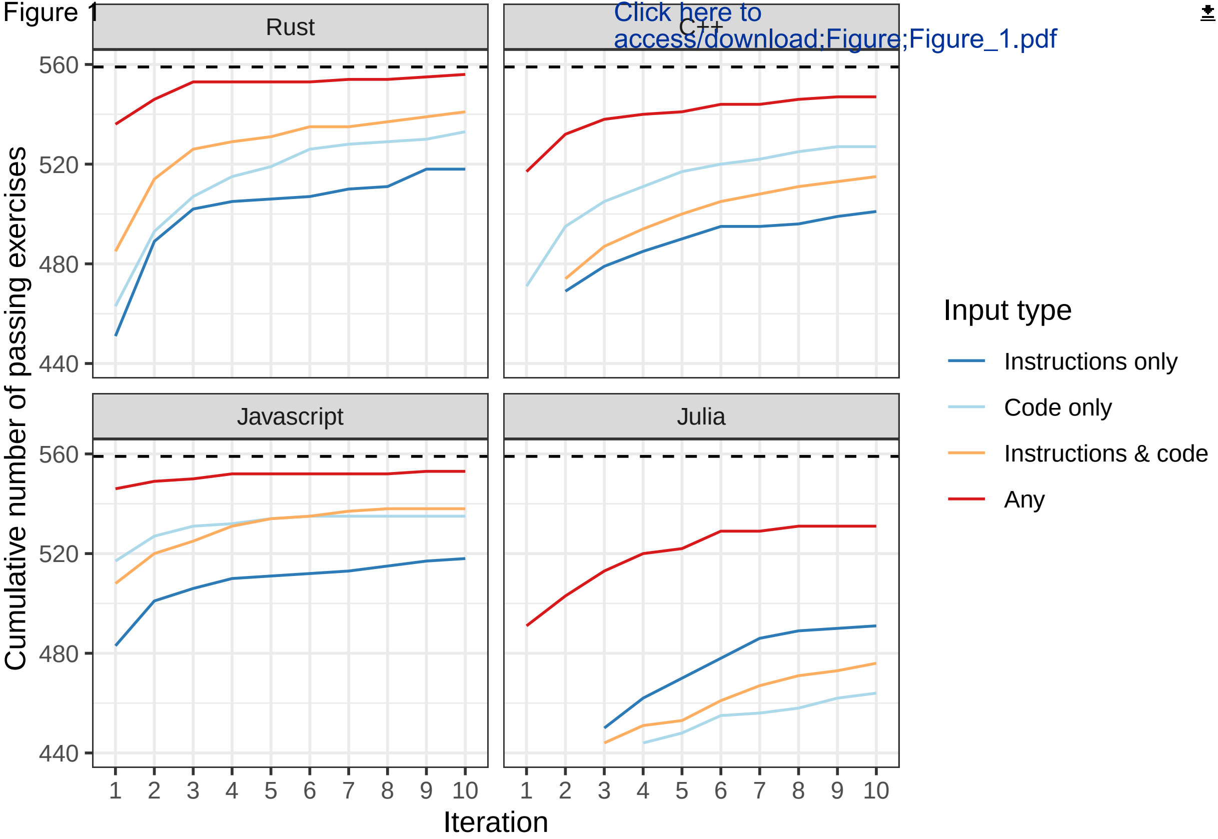

Figure 2

Click here to access/download;Figure;Figure_2.pdf

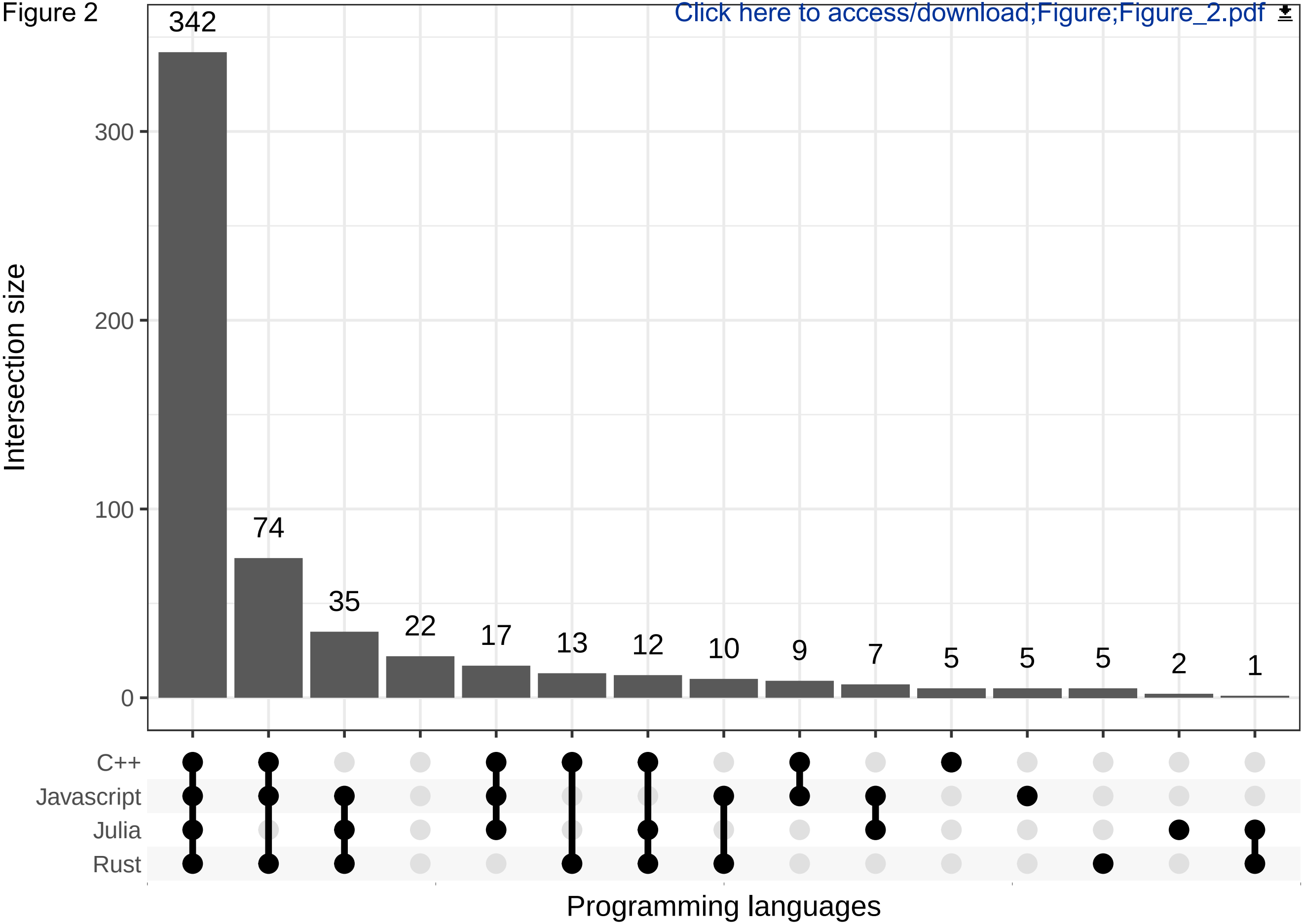

Figure 3

Click here to access/download;Figure;Figure_3.pdf

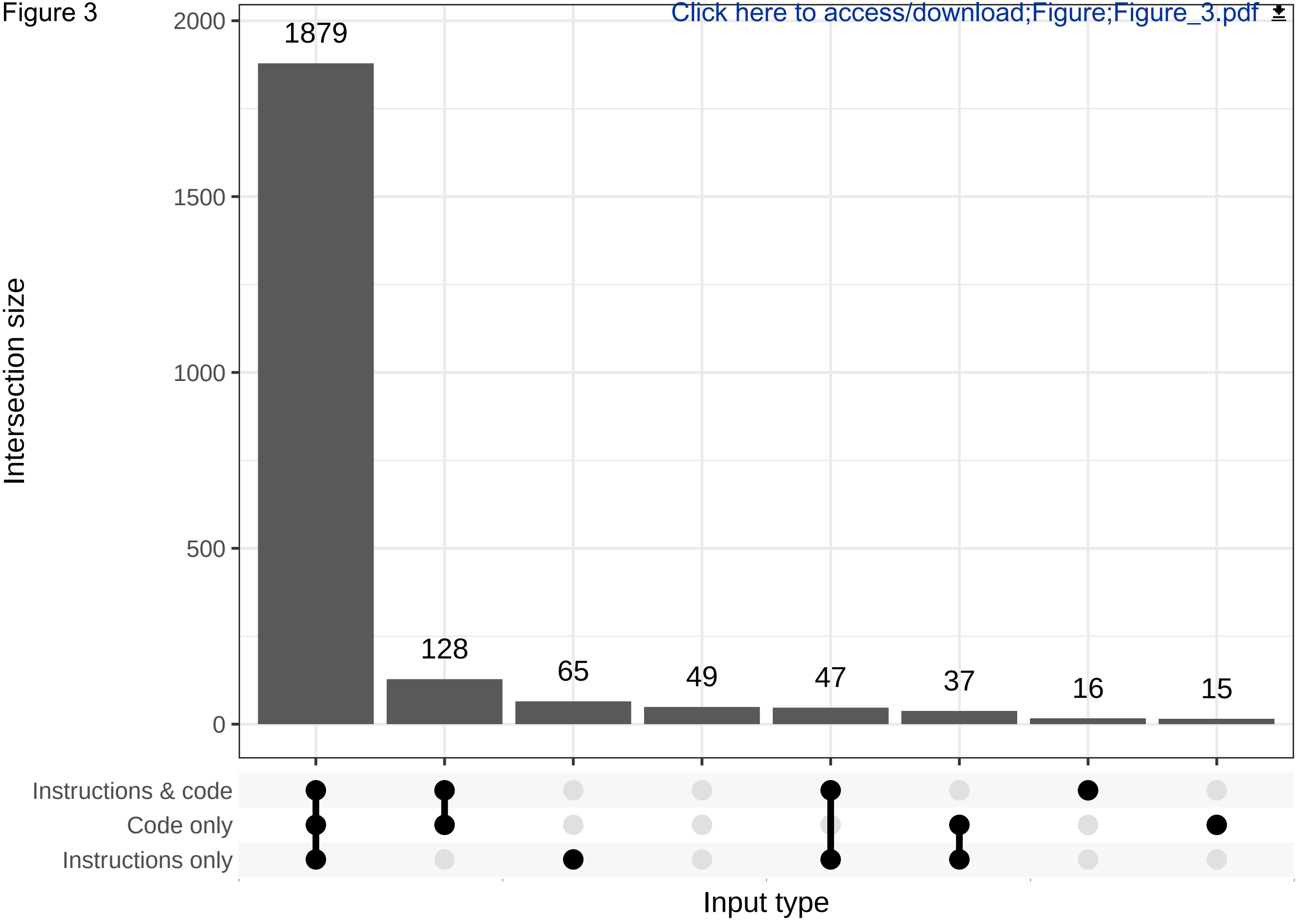

Figure 4

Click here to access/download;Figure;Figure_4.pdf

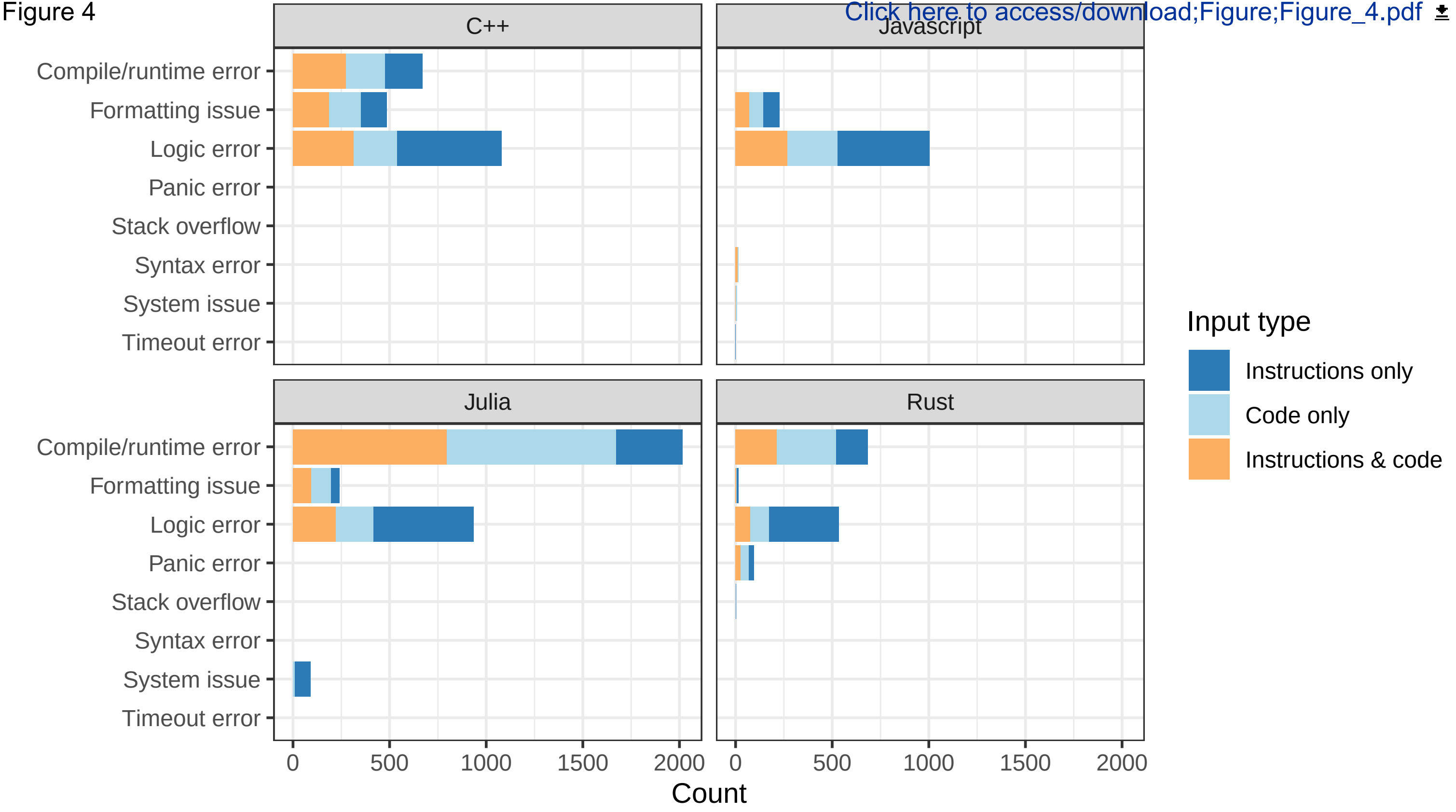

Figure 5

Click here to access/download;Figure;Figure_5.pdf

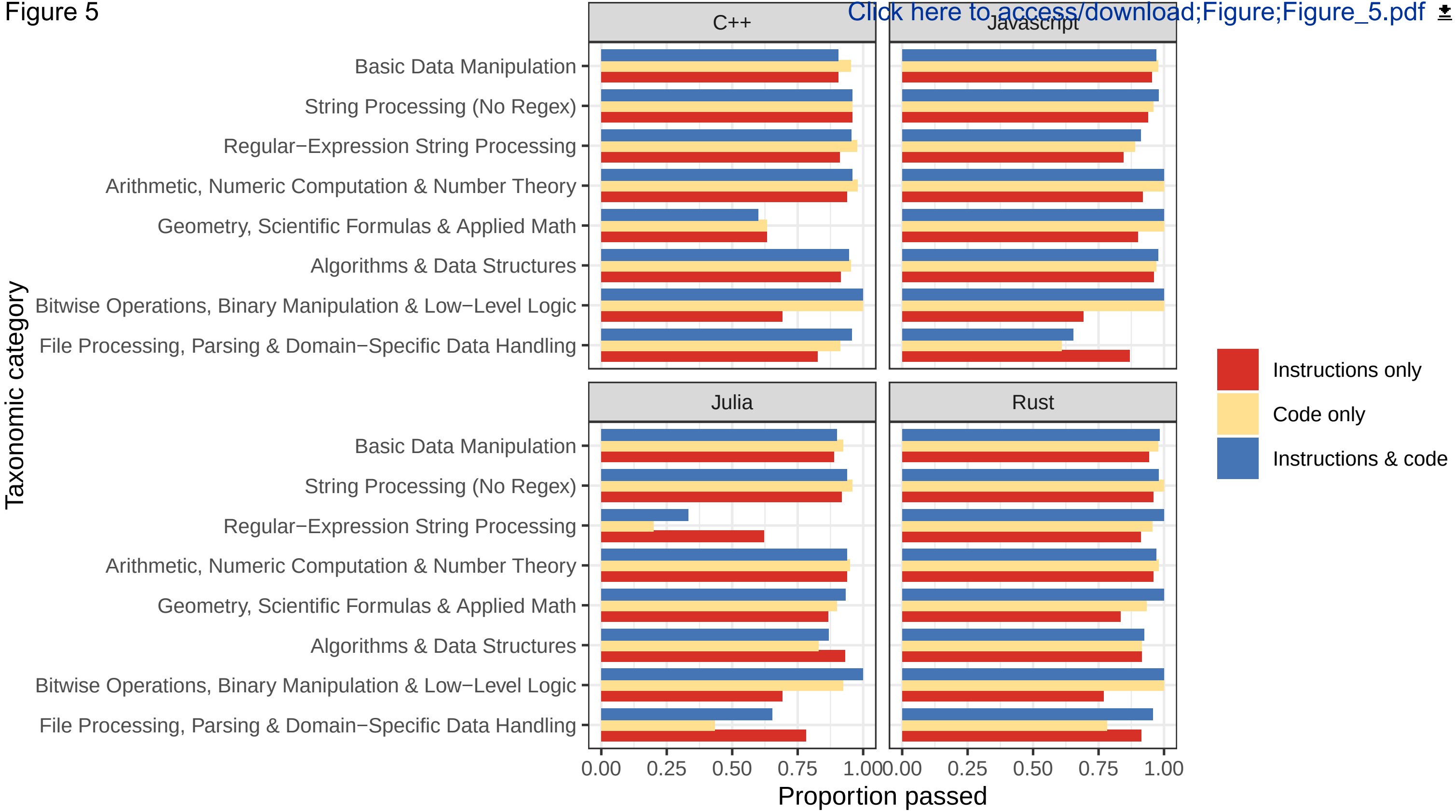

Figure 6

Click here to access/download;Figure;Figure_6.pdf

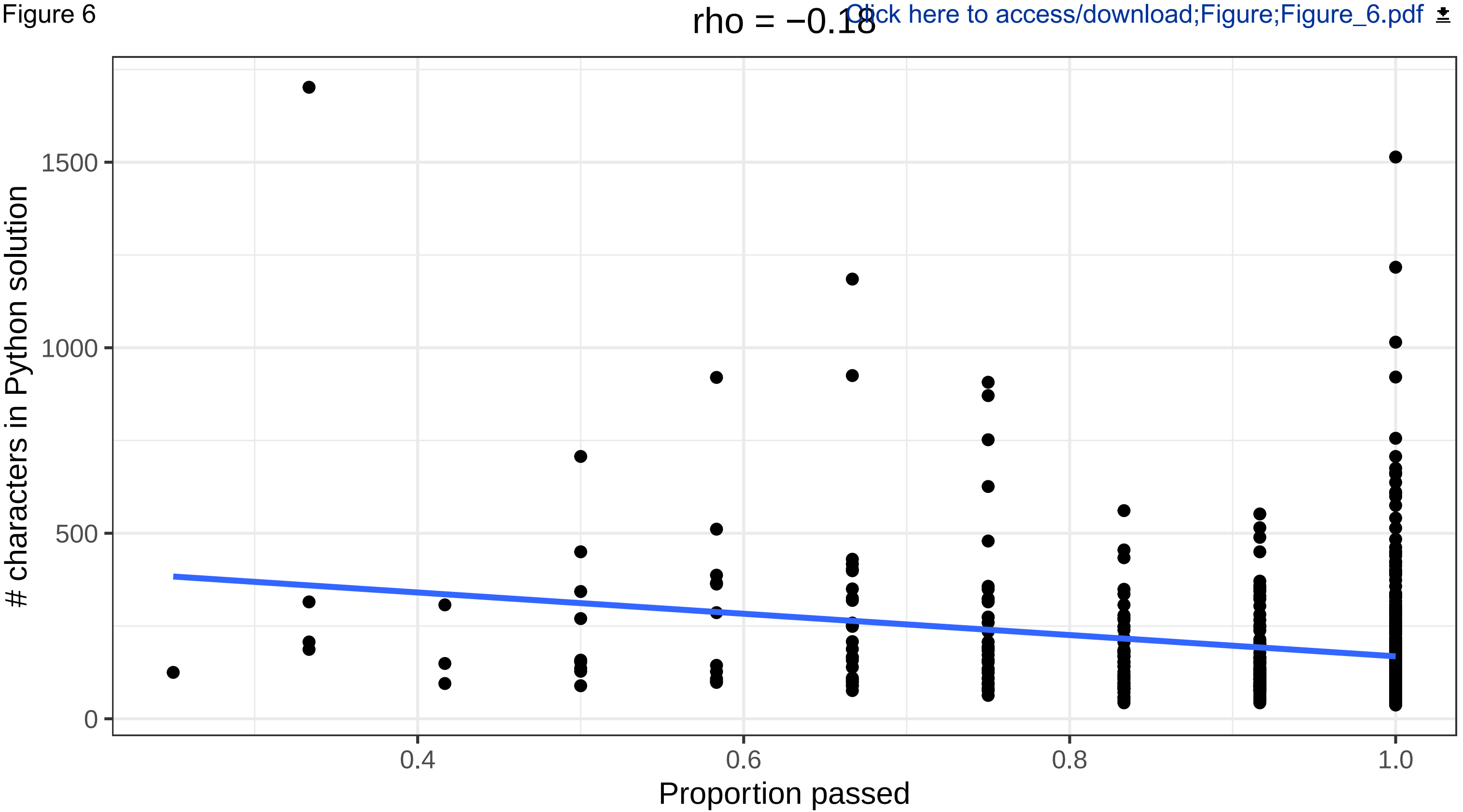

Figure 7

Click here to access/download;Figure;Figure_7.pdf

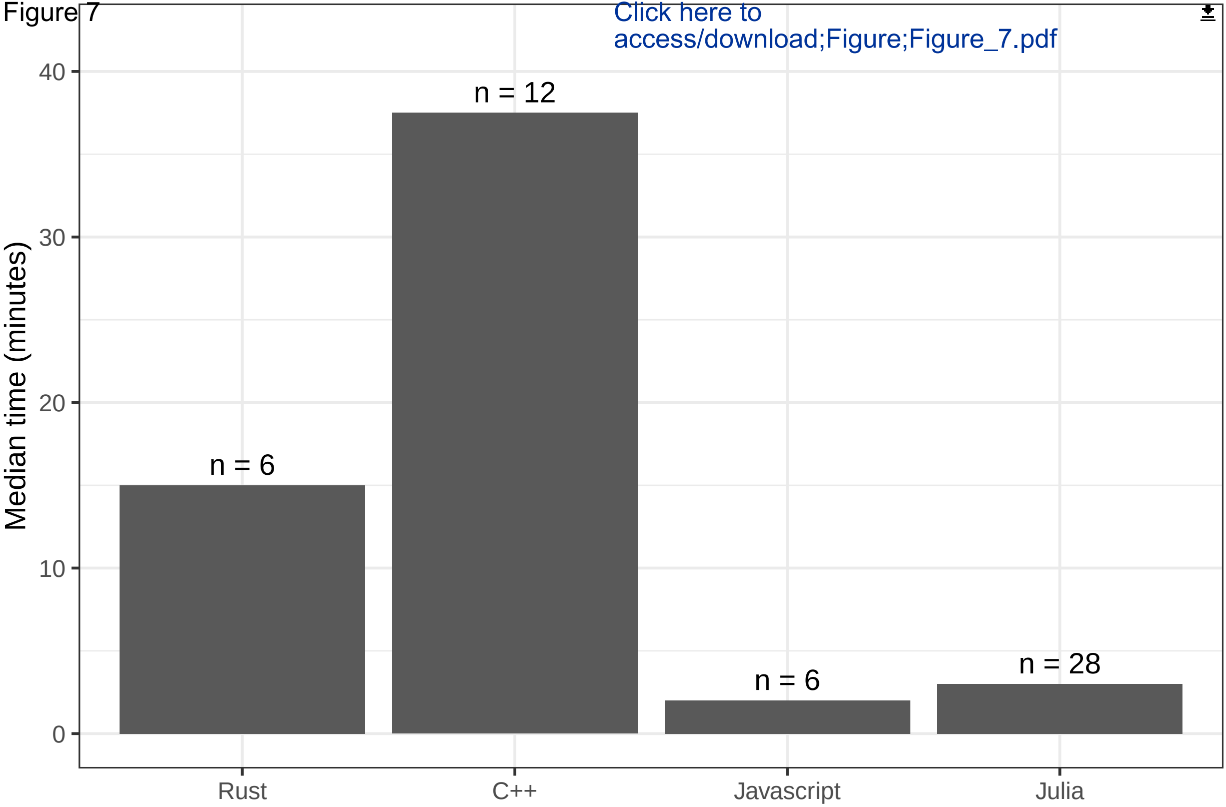

 Click here to access/download;Personal Cover;Reviewer Response.pdf

Thank you for taking time to review our manuscript entitled, "Translating short-form Python exercises to other programming languages using diverse prompting strategies." Below we provide a point-by-point response to the reviewers' comments. Our comments are in black and are bolded. The reviewers' comments are in gray.

We look forward to moving this manuscript forward to publication.

–--

Dear Dr. Piccolo,

Your manuscript "Translating short-form Python exercises to other programming languages using diverse prompting strategies" (GIGA-D-25-00265) has been assessed by our reviewers. Based on these reports, and my own assessment as Editor, I am pleased to inform you that it is potentially acceptable for publication in GigaScience, once you have carried out some essential revisions suggested by our reviewers.

Their reports, together with any other comments, are below….

Best wishes,

Hongfang Zhang
GigaScience
www.gigasciencejournal.com

Please include a point-by-point within the 'Response to Reviewers' box in the submission system. Please ensure you describe additional experiments that were carried out and include a detailed rebuttal of any criticisms or requested revisions that you disagreed with. Please also ensure that your revised manuscript conforms to the journal style, which can be found in the Instructions for Authors on the journal homepage. If the data and code has been modified in the revision process please be sure to update the public versions of this too.

Reviewer reports:

Reviewer #1: In this work, Piccolo and Stevens investigate the effectiveness of different strategies for translating code snippets across multiple programming languages using a large language model (GPT-4). The work provides valuable insights into how various prompting approaches affect translation accuracy across different language pairs. The paper is well written, includes appropriate figures and tables, and is practically relevant to the growing field of AI-assisted code development. I recommend publication after only minor revisions.

We thank the reviewer for taking the time to provide this review and for these positive comments.

The abstract states, "at least one strategy was successful for nearly every exercise." This phrasing may imply that there was a single strategy that was consistently effective, but I believe the authors mean to say that for each exercise, at least one of the strategies tested was effective. I recommend clarifying this ambiguity.

Thank you for this suggestion. We have updated this part of the Abstract according to this suggestion.

The authors mention that the code snippets considered are generally 20 lines or less, but there is no analysis of how translation accuracy varies with code length within this range. It would be valuable to examine whether 5-line snippets are translated more accurately than 20-line snippets, for example. If such analysis is beyond the current scope, the authors could at least acknowledge this limitation and suggest it for future work.

Thank you for this suggestion. We have added a figure and sentence to the results with an analysis of the relationship between Python code length and translation success. The relationship was statistically significant. (We used # of characters rather than # of lines as a surrogate for code complexity.)

Although the figures and tables provide a quantitative assessment of the results, the text itself often presents findings without specific metrics (e.g., percentages). This lack of specifics makes the text seem vague in places, even though the data is fully presented in the figures and tables. The authors shouldn't repeat all the figure/table values in the text, but mentioning specific metrics to highlight the most important findings might help make the text feel more precise. Here are specific instances where concrete percentages could replace vague language, to illustrate what I mean:

- "For Rust translations, performance improved rapidly in early iterations before beginning to plateau" (how much did it improve, perhaps in terms of percentage?)
- "Most commonly, translation was successful for all four languages" (how often exactly?)
- "It was also common for three of the four languages—in different combinations—to be successful" (how common, exactly?)
- "When translations were not successful for all input types, the most common scenarios were for translations to be successful either for A) code only and both inputs or B) instructions only" (how common?)

Thank you. We have added these percentages in the places mentioned.

In summary, this manuscript presents valuable research on code translation strategies with practical implications for AI-assisted programming. The experimental design is appropriate, and the findings are interesting. I recommend only minor revisions.

Thanks again!

Reviewer #2: This paper studies the effectiveness of ChatGPT4 in translating scripting code in Python into other languages. This is an increasingly common need: translating code across languages for education and research reproducibility.

The study works at a good scale—evaluating 559 exercises across four target languages.

The authors have shared data, scripts, and the CodeBuddy resource, which supports reproducibility. This is great!

Testing multiple prompting strategies (instructions only, code only, both) is a nice contribution. While this is common in NLP, it seems to be one of the first applications in this particular domain.

The writing is clear overall and the manuscript has a logical flow.

We thank the reviewer for taking the time to provide this review and for these positive comments.

Major concern:

The authors manually reviewed failed tests and counted some outputs as "qualitatively identical" even if tests failed due to syntax or formatting issues. The criteria for what counts as "qualitatively identical" are quite subjective. Other papers have used automated equivalence checking both output and semantics to reduce this subjectivity. Maybe at least mention it as a limitation.

Thank you. We now mention this as a limitation in the Discussion section.

Minor concerns:

Model Diversity: They only used GPT-4 and no other LLMs. While this is often expected in CS/NLP venues, it may be less of an issue here. The authors did discuss this as a limitation, so this is more of a minor point.

Thank you. We agree that this is a limitation and acknowledge that future work could address this.

Data Leakage: The exercises come from public sources. Although the authors state that no translated versions existed to their knowledge, GPT-4 may have been exposed to them during training since it was trained on data on the Internet. Maybe they should mention this in their limitation.

Thank you. We now address this limitation in the Discussion section.

Prompt Transparency: The prompts are described in the text, but it's unclear if they are shown verbatim. Usually, it's helpful to provide exact prompts as a figure or in the supplementary materials to improve reproducibility.

Thank you for this suggestion. We decided it would be best to provide the full prompt templates in the manuscript itself. As before, these templates have placeholders. Now they include both the user prompt and the system prompt for each scenario. We agree that this will support transparency.

Maybe use some more technical nlp terms? Right now their prompting strategy is called zero shot prompting, which is not mentioned at all.

Thank you. Because the journal's audience is broad and includes many who are not NLP/ML experts, we preferred to use less technical language. However, we agree that the term zero-shot prompting, in particular, is relevant because it conveys the idea that the models can be effective even when not trained for the specific types of tasks we were completing. We have modified the Introduction to use this term and explain this concept.

There is no mention of the types of exercises used or a table of benchmark characteristics (e.g., lines of code, number of functions). Adding a taxonomy of exercise types (string manipulation, recursion, I/O, etc.) would help readers understand how representative the dataset is. It would also be valuable to include summary statistics (average code length, complexity) and compare these to success rates. This would give much clearer insight into how complexity affects translation success. Maybe at least add a summary table of taxonomy of exercise types.

Thank you for this suggestion. We created definitions of exercise types and mapped each exercise to one of them. This is reported in the body of the manuscript and in a new figure. Additionally, we evaluated the relationship between code length (number of characters) as a simple surrogate for code complexity and found a statistically significant correlation. We also added a figure that addressed both of these topics.

For their retry strategy, the authors simply re-issued the same prompt up to 10 times without any additional feedback. It would be much more informative to experiment with richer feedback approaches like re-asking with the previous error message, or adding hints to see if these improve results. Maybe also mention it as a limitation.

Thank you. We now address this as a limitation in the Discussion section.
