# Supplementary material for: Translating short-form Python exercises to other programming languages using diverse prompting strategies
Source: Gigascience. 2025 Dec 8;14:giaf149. doi: 10.1093/gigascience/giaf149 (PMC12758376; doi:10.1093/gigascience/giaf149)
Supplement: giaf149_Authors_Response_To_Reviewer_Comments_Original_Submission [file giaf149_authors_response_to_reviewer_comments_original_submission.pdf]

Author's Response To Reviewer Comments

Thank you for taking time to review our manuscript entitled, "Translating short-form Python exercises to other programming languages using diverse prompting strategies." Below we provide a point-by-point response to the reviewers' comments.

We look forward to moving this manuscript forward to publication.

Dear Dr. Piccolo,

Your manuscript "Translating short-form Python exercises to other programming languages using diverse prompting strategies" (GIGA-D-25-00265) has been assessed by our reviewers. Based on these reports, and my own assessment as Editor, I am pleased to inform you that it is potentially acceptable for publication in GigaScience, once you have carried out some essential revisions suggested by our reviewers.

Their reports, together with any other comments, are below….

Best wishes,

Hongfang Zhang
GigaScience
www.gigasciencejournal.com

Please include a point-by-point within the 'Response to Reviewers' box in the submission system. Please ensure you describe additional experiments that were carried out and include a detailed rebuttal of any criticisms or requested revisions that you disagreed with. Please also ensure that your revised manuscript conforms to the journal style, which can be found in the Instructions for Authors on the journal homepage. If the data and code has been modified in the revision process please be sure to update the public versions of this too.

Reviewer reports:

Reviewer #1: In this work, Piccolo and Stevens investigate the effectiveness of different strategies for translating code snippets across multiple programming languages using a large language model (GPT-4). The work provides valuable insights into how various prompting approaches affect translation accuracy across different language pairs. The paper is well written, includes appropriate figures and tables, and is practically relevant to the growing field of AI-assisted code development. I recommend publication after only minor revisions.

> We thank the reviewer for taking the time to provide this review and for these positive comments.

The abstract states, "at least one strategy was successful for nearly every exercise." This phrasing may imply that there was a single strategy that was consistently effective, but I believe the authors mean to say that for each exercise, at least one of the strategies tested was effective. I recommend clarifying this ambiguity.

> Thank you for this suggestion. We have updated this part of the Abstract according to this suggestion.

The authors mention that the code snippets considered are generally 20 lines or less, but there is no analysis of how translation accuracy varies with code length within this range. It would be valuable to examine whether 5-line snippets are translated more accurately than 20-line snippets, for example. If such analysis is beyond the current scope, the authors could at least acknowledge this limitation and suggest it for future work.

> Thank you for this suggestion. We have added a figure and sentence to the results with an analysis of the relationship between Python code length and translation success. The relationship was statistically significant. (We used # of characters rather than # of lines as a surrogate for code complexity.)

Although the figures and tables provide a quantitative assessment of the results, the text itself often presents findings without specific metrics (e.g., percentages). This lack of specifics makes the text seem vague in places, even though the data is fully presented in the figures and tables. The authors shouldn't repeat all the figure/table values in the text, but mentioning specific metrics to highlight the most important findings might help make the text feel more precise. Here are specific instances where concrete percentages could replace vague language, to illustrate what I mean:

- "For Rust translations, performance improved rapidly in early iterations before beginning to plateau" (how much did it improve, perhaps in terms of percentage?)
- "Most commonly, translation was successful for all four languages" (how often exactly?)
- "It was also common for three of the four languages—in different combinations—to be successful" (how common, exactly?)
- "When translations were not successful for all input types, the most common scenarios were for translations to be successful either for A) code only and both inputs or B) instructions only" (how common?)

> Thank you. We have added these percentages in the places mentioned.

In summary, this manuscript presents valuable research on code translation strategies with practical implications for AI-assisted programming. The experimental design is appropriate, and the findings are interesting. I recommend only minor revisions.

> Thanks again!

Reviewer #2: This paper studies the effectiveness of ChatGPT4 in translating
scripting code in Python into other languages. This is an increasingly common need: translating code across languages for education and research reproducibility.

The study works at a good scale—evaluating 559 exercises across four
target languages.

The authors have shared data, scripts, and the CodeBuddy resource, which supports reproducibility. This is great!

Testing multiple prompting strategies (instructions only, code only, both) is a nice contribution. While this is common in NLP, it seems to be one of the first applications in this particular domain.

The writing is clear overall and the manuscript has a logical flow.

> We thank the reviewer for taking the time to provide this review and for these positive comments.

Major concern:

The authors manually reviewed failed tests and counted some outputs as "qualitatively identical" even if tests failed due to syntax or formatting issues. The criteria for what counts as "qualitatively identical" are quite subjective. Other papers have used automated equivalence checking both output and semantics to reduce this subjectivity. Maybe at least mention it as a limitation.

> Thank you. We now mention this as a limitation in the Discussion section.

Minor concerns:

Model Diversity: They only used GPT-4 and no other LLMs. While this is often expected in CS/NLP venues, it may be less of an issue here. The authors did discuss this as a limitation, so this is more of a minor point.

> Thank you. We agree that this is a limitation and acknowledge that future work could address this.

Data Leakage: The exercises come from public sources. Although the authors state that no translated versions existed to their knowledge, GPT-4 may have been exposed to them during training since it was trained on data on the Internet. Maybe they should mention this in their limitation.

> Thank you. We now address this limitation in the Discussion section.

Prompt Transparency: The prompts are described in the text, but it's unclear if they are shown verbatim. Usually, it's helpful to provide exact prompts as a figure or in the supplementary materials to improve reproducibility.

> Thank you for this suggestion. We decided it would be best to provide the full prompt templates in the manuscript itself. As before, these templates have placeholders. Now they include both the user prompt and the system prompt for each scenario. We agree that this will support transparency.

Maybe use some more technical nlp terms? Right now their prompting strategy is called zero shot prompting, which is not mentioned at all.

> Thank you. Because the journal's audience is broad and includes many who are not NLP/ML experts, we preferred to use less technical language. However, we agree that the term zero-shot prompting, in particular, is relevant because it conveys the idea that the models can be effective even when not trained for the specific types of tasks we were completing. We have modified the Introduction to use this term and explain this concept.

There is no mention of the types of exercises used or a table of benchmark characteristics (e.g., lines of code, number of functions). Adding a taxonomy of exercise types (string manipulation, recursion, I/O, etc.) would help readers understand how representative the dataset is. It would also be valuable to include summary statistics (average code length, complexity) and compare these to success rates. This would give much clearer insight into how complexity affects translation success. Maybe at least add a summary table of taxonomy of exercise types.

> Thank you for this suggestion. We created definitions of exercise types and mapped each exercise to one of them. This is reported in the body of the manuscript and in a new figure. Additionally, we evaluated the relationship between code length (number of characters) as a simple surrogate for code complexity and found a statistically significant correlation. We also added a figure that addressed both of these topics.

For their retry strategy, the authors simply re-issued the same prompt up to 10 times without any additional feedback. It would be much more informative to experiment with richer feedback approaches like re-asking with the previous error message, or adding hints to see if these improve results. Maybe also mention it as a limitation.

> Thank you. We now address this as a limitation in the Discussion section.

Cookie Preference Center

We use cookies which are necessary to make our site work. We may also use additional cookies to analyze, improve and personalize our content and your digital experience. For more information, see our Cookie Policy.

You may choose not to allow some types of cookies. However, blocking some types may impact your experience of our site and the services we are able to offer. See the different category headings below to find out more or change your settings.

Allow all

Manage Consent Preferences

Strictly Necessary Cookies

Always active

These cookies are necessary for the website to function and cannot be switched off in our systems. They are usually only set in response to actions made by you which amount to a request for services, such as setting your privacy preferences, logging in or filling in forms. You can set your browser to block or alert you about these cookies, but some parts of the site will not then work.

Functional Cookies

These cookies enable the website to provide enhanced functionality and personalisation. They may be set by us or by third party providers whose services we have added to our pages. If you do not allow these cookies then some or all of these services may not function properly.

Performance Cookies

These cookies allow us to count visits and traffic sources so we can measure and improve the performance of our site. They help us to know which pages are the most and least popular and see how visitors move around the site.

Targeting Cookies

These cookies may be set through our site by our advertising partners. They may be used by those companies to build a profile of your interests and show you relevant adverts on other sites. If you do not allow these cookies, you will experience less targeted advertising.

Cookie List

Clear

Apply Cancel
Consent Leg.Interest

Confirm my choices

Powered by onetrus
