# Supplementary material for: Translating short-form Python exercises to other programming languages using diverse prompting strategies
Source: Gigascience. 2025 Dec 8;14:giaf149. doi: 10.1093/gigascience/giaf149 (PMC12758376; doi:10.1093/gigascience/giaf149)
Supplement: giaf149_Reviewer_1_Report_Original_Submission — Jacob Durrant -- 8/4/2025 [file giaf149_reviewer_1_report_original_submission.pdf]

Reviewer Report

Title: Translating short-form Python exercises to other programming languages using diverse prompting strategies

Version: Original Submission **Date:** 8/4/2025

Reviewer name: Jacob Durrant

Reviewer Comments to Author:

In this work, Piccolo and Stevens investigate the effectiveness of different strategies for translating code snippets across multiple programming languages using a large language model (GPT-4). The work provides valuable insights into how various prompting approaches affect translation accuracy across different language pairs. The paper is well written, includes appropriate figures and tables, and is practically relevant to the growing field of AI-assisted code development. I recommend publication after only minor revisions.

The abstract states, "at least one strategy was successful for nearly every exercise." This phrasing may imply that there was a single strategy that was consistently effective, but I believe the authors mean to say that for each exercise, at least one of the strategies tested was effective. I recommend clarifying this ambiguity.

The authors mention that the code snippets considered are generally 20 lines or less, but there is no analysis of how translation accuracy varies with code length within this range. It would be valuable to examine whether 5-line snippets are translated more accurately than 20-line snippets, for example. If such analysis is beyond the current scope, the authors could at least acknowledge this limitation and suggest it for future work.

Although the figures and tables provide a quantitative assessment of the results, the text itself often presents findings without specific metrics (e.g., percentages). This lack of specifics makes the text seem vague in places, even though the data is fully presented in the figures and tables. The authors shouldn't repeat all the figure/table values in the text, but mentioning specific metrics to highlight the most important findings might help make the text feel more precise. Here are specific instances where concrete percentages could replace vague language, to illustrate what I mean:

- "For Rust translations, performance improved rapidly in early iterations before beginning to plateau" (how much did it improve, perhaps in terms of percentage?)
- "Most commonly, translation was successful for all four languages" (how often exactly?)
- "It was also common for three of the four languagesâ€”in different combinationsâ€”to be successful" (how common, exactly?)
- "When translations were not successful for all input types, the most common scenarios were for translations to be successful either for A) code only and both inputs or B) instructions only" (how common?)

In summary, this manuscript presents valuable research on code translation strategies with practical implications for AI-assisted programming. The experimental design is appropriate, and the findings are interesting. I recommend only minor revisions.

Methods

Are the methods appropriate to the aims of the study, are they well described, and are necessary controls included? Choose an item.

Conclusions

Are the conclusions adequately supported by the data shown? Choose an item.

Reporting Standards

Does the manuscript adhere to the journal's guidelines on minimum standards of reporting? Choose an item.

Choose an item.

Statistics

Are you able to assess all statistics in the manuscript, including the appropriateness of statistical tests used? Choose an item.

Quality of Written English

Please indicate the quality of language in the manuscript: Choose an item.

Declaration of Competing Interests

Please complete a declaration of competing interests, considering the following questions:

- Have you in the past five years received reimbursements, fees, funding, or salary from an organisation that may in any way gain or lose financially from the publication of this manuscript, either now or in the future?
- Do you hold any stocks or shares in an organisation that may in any way gain or lose financially from the publication of this manuscript, either now or in the future?
- Do you hold or are you currently applying for any patents relating to the content of the manuscript?
- Have you received reimbursements, fees, funding, or salary from an organization that holds or has applied for patents relating to the content of the manuscript?
- Do you have any other financial competing interests?
- Do you have any non-financial competing interests in relation to this paper?

If you can answer no to all of the above, write 'I declare that I have no competing interests' below. If your reply is yes to any, please give details below.

I declare that I have no competing interests.

I agree to the open peer review policy of the journal. I understand that my name will be included on my report to the authors and, if the manuscript is accepted for publication, my named report including any attachments I upload will be posted on the website along with the authors' responses. I agree for my report to be made available under an Open Access Creative Commons CC-BY license (http://creativecommons.org/licenses/by/4.0/). I understand that any comments which I do not wish to be included in my named report can be included as confidential comments to the editors, which will not be published.

Choose an item.

To further support our reviewers, we have joined with Publons, where you can gain additional credit to further highlight your hard work (see: https://publons.com/journal/530/gigascience). On publication of this paper, your review will be automatically added to Publons, you can then choose whether or not to claim your Publons credit. I understand this statement.

Yes Choose an item.
