# Supplementary material for: Translating short-form Python exercises to other programming languages using diverse prompting strategies
Source: Gigascience. 2025 Dec 8;14:giaf149. doi: 10.1093/gigascience/giaf149 (PMC12758376; doi:10.1093/gigascience/giaf149)
Supplement: giaf149_Reviewer_2_Report_Original_Submission — C. Titus Brown -- 8/10/2025 [file giaf149_reviewer_2_report_original_submission.pdf]

Reviewer Report

Title: Translating short-form Python exercises to other programming languages using diverse prompting strategies

Version: Original Submission **Date:** 8/10/2025

Reviewer name: C. Titus Brown

Reviewer Comments to Author:

This paper studies the effectiveness of ChatGPT4 in translating scripting code in Python into other languages. This is an increasingly common need: translating code across languages for education and research reproducibility.
The study works at a good scaleâ€"evaluating 559 exercises across four target languages
The authors have shared data, scripts, and the CodeBuddy resource, which supports reproducibility. This is great!
Testing multiple prompting strategies (instructions only, code only, both) is a nice contribution. While this is common in NLP, it seems to be one of the first applications in this particular domain.
The writing is clear overall and the manuscript has a logical flow.
Major concern:
The authors manually reviewed failed tests and counted some outputs as "qualitatively identical" even if tests failed due to syntax or formatting issues. The criteria for what counts as "qualitatively identical" are quite subjective. Other papers have used automated equivalence checking both output and semantics to reduce this subjectivity. Maybe at least mention it as a limitation.
Minor concerns:
Model Diversity: They only used GPT-4 and no other LLMs. While this is often expected in CS/NLP venues, it may be less of an issue here. The authors did discuss this as a limitation, so this is more of a minor point.
Data Leakage: The exercises come from public sources. Although the authors state that no translated versions existed to their knowledge, GPT-4 may have been exposed to them during training since it was trained data on Internet. Maybe they should mention this in their limitation.
Prompt Transparency: The prompts are described in text, but it's unclear if they are shown verbatim. Usually, it's helpful to provide exact prompts as a figure or in the supplementary materials to improve

reproducibility.
Maybe use some more technical nlp terms? Right now their prompting strategy is called zero shot prompting, which is not mentioned at all.
There is no mention of the types of exercises used or a table of benchmark characteristics (e.g., lines of code, number of functions). Adding a taxonomy of exercise types (string manipulation, recursion, I/O, etc.) would help readers understand how representative the dataset is. It would also be valuable to include summary statistics (average code length, complexity) and compare these to success rates. This would give much clearer insight into how complexity affects translation success. Maybe at least add a summary table of taxonomy of exercise types.
For their retry strategy, the authors simply re-issued the same prompt up to 10 times without any additional feedback. It would be much more informative to experiment with richer feedback approaches like re-asking with the previous error message, or adding hints to see if these improve results. Maybe also mention it as limitation.

Methods

Are the methods appropriate to the aims of the study, are they well described, and are necessary controls included? Choose an item.

Conclusions

Are the conclusions adequately supported by the data shown? Choose an item.

Reporting Standards

Does the manuscript adhere to the journal's guidelines on minimum standards of reporting? Choose an item.

Choose an item.

Statistics

Are you able to assess all statistics in the manuscript, including the appropriateness of statistical tests used? Choose an item.

Quality of Written English

Please indicate the quality of language in the manuscript: Choose an item.

Declaration of Competing Interests

Please complete a declaration of competing interests, considering the following questions:

- Have you in the past five years received reimbursements, fees, funding, or salary from an organisation that may in any way gain or lose financially from the publication of this manuscript, either now or in the future?
- Do you hold any stocks or shares in an organisation that may in any way gain or lose financially from the publication of this manuscript, either now or in the future?
- Do you hold or are you currently applying for any patents relating to the content of the manuscript?
- Have you received reimbursements, fees, funding, or salary from an organization that holds or has applied for patents relating to the content of the manuscript?
- Do you have any other financial competing interests?
- Do you have any non-financial competing interests in relation to this paper?

If you can answer no to all of the above, write 'I declare that I have no competing interests' below. If your reply is yes to any, please give details below.

I declare that I have no competing interests.

I agree to the open peer review policy of the journal. I understand that my name will be included on my report to the authors and, if the manuscript is accepted for publication, my named report including any attachments I upload will be posted on the website along with the authors' responses. I agree for my report to be made available under an Open Access Creative Commons CC-BY license (http://creativecommons.org/licenses/by/4.0/). I understand that any comments which I do not wish to be included in my named report can be included as confidential comments to the editors, which will not be published.

Choose an item.

To further support our reviewers, we have joined with Publons, where you can gain additional credit to further highlight your hard work (see: https://publons.com/journal/530/gigascience). On publication of this paper, your review will be automatically added to Publons, you can then choose whether or not to claim your Publons credit. I understand this statement.

Yes Choose an item.
